# Disturbed metabolic adaptation drives natural killer cell dysfunction in association with nosocomial infection during human sepsis

**DOI:** 10.1016/j.ebiom.2026.106345

**Published:** 2026-06-26

**Authors:** André van der Wurff, Monika Gambusz, Bettina Budeus, Maren Claus, Carsten Watzl, Dani Miteva, Brendon P. Scicluna, Ignacio Rubio, Elena Siakaeva, Frederic Zinsmeister, Sonja Vonderhagen, Nadja Klenke, Frank Herbstreit, Karsten Schmidt, Marc M. Berger, Thorsten Brenner, Marcel Dudda, Stefanie B. Flohé

**Affiliations:** a Department of Trauma, Hand, and Reconstructive Surgery, University Hospital Essen, University Duisburg-Essen, Essen, Germany; b Genomics and Transcriptomics Facility, Institute of Cell Biology, University Hospital Essen, Essen, Germany; c Leibniz Research Center for Working Environment and Human Factors at TU Dortmund (IfADo), Dortmund, Germany; d Department of Applied Biomedical Science, Faculty of Health Sciences, University of Malta, Msida, Malta; e Centre for Molecular Medicine and Biobanking, Biomedical Sciences bldg., University of Malta, Msida, Malta; f Department for Anesthesiology & Intensive Care Medicine, Jena University Hospital, Jena, Germany; g Department of Anesthesiology and Intensive Care Medicine, University Hospital Essen, University Duisburg-Essen, Essen, Germany; h Department of Orthopedics and Trauma Surgery, BG-Klinikum Duisburg, Duisburg, Germany

**Keywords:** Sepsis, Immunosuppression, Natural killer cells, Metabolism, Mammalian target of rapamycin C1, Interferon γ

## Abstract

**Background:**

Patients with sepsis are highly susceptible to detrimental nosocomial infections. During bacterial infection, natural killer (NK) cells release Interferon (IFN) γ that drives the elimination of invading pathogens. Interleukin (IL) 12 in synergy with other cytokines increases sensing and uptake of nutrients by NK cells for metabolic adaptation required for induction of IFN-γ production. We hypothesised that inappropriate function of NK cells was associated with nosocomial infections during human sepsis and linked to altered metabolic adaptation.

**Methods:**

We performed a longitudinal exploratory study on circulating human NK cells during sepsis and evaluated adaptation of nutrient sensing, activation of the metabolic hub mammalian target of rapamycin (mTOR) C1, and IFN-γ production upon exposure to *Staphylococcus* aureus as a model for an opportunistic pathogen *in vitro*. The involvement of cell-intrinsic and extrinsic pathways in NK cell function was addressed.

**Findings:**

Expression of the IL-12 receptor (p < 0.001) and downstream production of IFN-γ (p < 0.01) after exposure to *S. aureus* were suppressed in NK cells for at least 14 days after sepsis diagnosis, particularly in patients who developed secondary infections (p < 0.01). Mechanistically, suppression of NK cells was independent from environmental cues but was cell-intrinsic and associated with impaired activation of mTORC1 and with reduced expression of nutrient transporters required for anabolic metabolism. Inhibition of AMP kinase (AMPK) restored mTORC1 activity (p < 0.01) and increased the production of IFN-γ (p < 0.01) in NK cells from septic patients.

**Interpretation:**

Defective metabolic regulation is associated with persistent NK cell dysfunction during human sepsis and might represent a potential therapeutic target to improve immune competence and decrease the risk for nosocomial infections.

**Funding:**

The study was supported by the “Research and Training” program “ELAN” for medical students of the medical faculty of the University Duisburg-Essen.

**Research in context**

**Evidence before this study**

Sepsis is a life-threatening systemic infection that is mediated by a dysregulated host response to the pathogen. Sepsis is a major cause of death world-wide and represents a global health burden. Patients surviving the acute sepsis episode that is characterised by overwhelming inflammation and organ failure face an increased susceptibility to detrimental, frequently bacterial, secondary infections due to the development of long-lasting immunosuppression which is only incompletely understood. In case of bacterial infection, natural Killer (NK) cells as part of the innate immune system are stimulated by Interleukin (IL) 12 and in turn secrete large amounts of Interferon (IFN) γ that promotes the clearance of pathogens by other immune cells. Activated NK cells require metabolic adaptation to cope with the increased demand of energy and newly synthesised biomolecules. Early after sepsis diagnosis, NK cells are severely compromised in their ability to produce IFN-γ. To date, it is unclear whether this dysfunction of NK cells is a phenomenon of very early sepsis or whether it persists and may contribute to the enhanced risk for secondary infections. The underlying pathomechanisms that might point to potential therapeutic targets have not been addressed so far.

**Added value of this study**

In this longitudinal exploratory study on NK cells from patients with sepsis (from 1 d to 28 d after sepsis diagnosis) we detected a persistent defect in IFN-γ production in response to *Staphylococcus aureus* as a putative secondary infectious insult especially in patients who later developed nosocomial infections. This impairment was independent from environmental cues but was associated with a cell-intrinsic modulation of metabolic regulation including reduced expression of nutrient transporters and suppressed activity of “mammalian target of rapamycin” (mTORC1), the key hub in cellular metabolic adaptation. Mechanistically, pharmacological inhibition of AMP-induced kinase (AMPK) restored mTORC1 signalling and IFN-γ production suggesting that a disturbed metabolic balance contributes to NK cell dysfunction during sepsis.

**Implications of all the available evidence**

In summary, our study identifies defective metabolic regulation as an explanation for previously unrecognised mechanisms of NK cell dysfunction during human sepsis that might represent a potential therapeutic target to improve immune competence of septic patients and decrease the risk for nosocomial infections.

## Introduction

Sepsis is defined as life-threatening organ dysfunction caused by a dysregulated host response to infection.^1^ In 2021, 166 million cases of sepsis and 21 million sepsis-related deaths were reported world-wide, which makes sepsis responsible for almost one third of all deaths.^2^

Although many types of micro-organisms can trigger sepsis, most cases are caused by bacteria.^3^ The pathogen-induced activation of the immune system triggers both pro- and anti-inflammatory pathways.^4^ Overwhelming inflammation combined with ensuing haemodynamic anomalies promotes organ dysfunction that is associated with an increased risk of early death.^5^ Patients surviving the acute sepsis episode face an increased susceptibility to life-threatening secondary infections and viral reactivation due to the development of long-lasting immunosuppression.^6^ *Staphylococcus aureus*, *E. coli*, and *Candida albicans* are known causative pathogens of secondary infections in septic patients with varying incidence depending on the infection focus.^7^^,^^8^ The pathomechanisms underlying the development of immunosuppression are still incompletely understood.^9^^,^^10^

Natural Killer (NK) cells are innate immune cells involved in the defence against viral and bacterial infections or in the eradication of tumour cells.^11^ Two major subpopulations of human circulating NK cells are distinguished: CD56^dim^ NK cells possess high cytotoxic capacity and mainly support the elimination of tumour cells and cells infected by viruses which is driven by direct engagement of so called “activating receptors” on NK cells by tumour and virus-infected cells. CD56^bright^ NK cells that represent 10% of circulating NK cells are potent in the release of cytokines and chemokines and thereby interact with other immune cells. CD56^bright^ NK cells are activated by innate cytokines such as Interleukin (IL) 12, IL-15, or IL-18 which are secreted by “accessory cells” such as dendritic cells (DCs) and monocytes/macrophages upon encountering invading pathogens.^11^^,^^12^ In turn, NK cells release Interferon (IFN)γ that further stimulates the release of cytokines by DCs creating a positive feedback loop.^13^ Moreover, NK cell-derived IFN-γ activates phagocytes for the elimination of bacteria.^12^

IL-12 engages the IL-12 receptor on NK cells that consists of the β1 chain and the signal-transducing β2 chain (IL-12Rβ2).^14^ IL-12R signalling induces the transcription of the *IFNG* gene.^15^^,^^16^ IL-18 and IL-15 synergise with IL-12 for increased IFN-γ synthesis.^17^ On the other hand, regulatory cytokines, such as Transforming growth factor (TGF) β, suppress the release of IFN-γ by NK cells.^18^

In addition, the function of NK cells is critically dependent on their cellular metabolism. Upon stimulation, human NK cells undergo metabolic reprogramming and increase the rate of oxidative phosphorylation and glycolysis to rapidly generate energy for the production of effector molecules such as IFN-γ.^19^^,^^20^ To cope with the increased demand of nutrients, NK cells increase the expression of diverse transporters or receptors including the transferrin receptor CD71 (iron uptake), glucose transporter (GLUT) 1, and the essential L-type amino acid transporter (LAT1).^21^^,^^22^

The key hub of metabolic reprogramming is the mammalian target of rapamycin (mTOR) complex 1 (mTORC1) that is activated in the presence of sufficient nutrient availability and required for NK cell effector function.^23^ As an example, the uptake of large amino acids via LAT1/CD98 activates mTORC1.^24^ Consequently, mTORC1 promotes the expression of other nutrient transporters such as CD71, GLUT1, components of the glycolytic pathway, and fosters protein synthesis, cell growth, and proliferation.^20^^,^^24^ mTORC1 also promotes the production of IFN-γ by CD56^bright^ NK cells.^22^ The activation of mTORC1 in NK cells is negatively regulated by TGF-β^25^ and positively regulated by IL-15.^26^^,^^27^ Perhaps the most important cellular regulator of mTORC1 is the adenosine monophosphate kinase (AMPK), which usually acts as a functional counterpart of mTORC1. AMPK is activated under nutrient/energy-deprived conditions and mediates the switch from anabolic to catabolic metabolism.^28^ Among other targets, AMPK downregulates mTORC1 activity via the phosphorylation of the tuberous sclerosis complex (TSC) complex, the immediate upstream regulator of mTORC1.^29^

Conflicting results exist on the role of NK cells in sepsis.^30^^,^^31^ In animal models of sepsis, NK cells play a detrimental role as they contribute to early hyperinflammation, organ damage, and morbidity^32^^,^^33^ presumably through the release of IFN-γ. Whether NK cells likewise release IFN-γ during human sepsis *in vivo* is unknown so far. Controversial data exist on the reactivity of NK cells from septic patients to stimulation with microbial components *in vitro* (as a correlate to their responsiveness to a secondary infectious insult) showing increased and reduced production of IFN-γ.^34^^,^^35^ The kinetics of the altered NK cell function during the course of sepsis and the underlying mechanisms have not been elucidated so far.

Considering the relevance of the cellular metabolism in NK cell function and given the well-established metabolic derangements in human sepsis,^36^ we hypothesised that changes in metabolic reprogramming contribute to NK cell dysfunction during severe systemic infection. In the present exploratory study, we comprehensively investigated the function of NK cells within circulating leukocytes during the first 28 d after sepsis diagnosis at baseline and in response to a secondary bacterial challenge with *S. aureus* as cause of secondary infections at diverse sites in septic patients.^7^^,^^8^ We provide evidence for a profound, long-lasting, cell-intrinsic deterioration of nutrient sensing and an imbalance of mTORC1/AMPK as a putative target for restoration of NK cell function during sepsis.

## Methods

### Patients and study design

Inclusion criteria for this exploratory study were an acute sepsis according to the sepsis-3 criteria and age ≥18 years. Organ dysfunction, defined as a change in the SOFA score of ≥2 points in addition to suspected or documented infection, led to the inclusion of the patients. Male and female subjects (self-reported and recorded in medical records) were included. Sex-dependent differences were not addressed. Patients and control subjects were recruited from Western Germany and were of European ancestry. Participants and controls were not asked to self-identify their race or ethnicity. Infections were identified by clinical manifestations as well as laboratory tests and radiological examinations and were confirmed by a positive microbiological assessment of blood, sputum, bronchoalveolar lavage and wound swabs. Excluded were patients with active or prior cancer, any type of chemotherapy, prior sepsis, prehospital immunosuppressants, severe autoimmunity, viral hepatitis, HIV infection, congenital immunodeficiency, underlying haematological disease, metformin therapy, and age <18 years. Secondary infection was defined as any new-onset infection (diagnosed by blood culture or bronchoalveolar lavage and being caused by microorganisms distinct to the sepsis-inducing agent) which started beyond 48 h after diagnosis of the primary, sepsis-inducing infection and, therefore, was associated with a relevant change in anti-infective therapy.^7^ The study followed the principles of the Helsinki declaration, and was reviewed and approved by the ethics committee of the University Hospital Essen (20-9352-BO). Informed consent was obtained from patients or their legal representatives.

Due to the exploratory nature of our study we did not perform a priori power analysis. According to our previous work on NK cells from non-septic, critically ill patients^37^^,^^38^ we estimated that the size of n = 20 for each group should be appropriate for our study. Patients who were admitted to the intensive care units of two departments (Department of Anaesthesiology and Intensive Care Medicine and Department of Trauma, Hand, and Reconstructive Surgery) at the University Hospital Essen were consecutively screened for eligibility between March and October 2021. All patients who met the criteria were included in the study unless laboratory capacity was exceeded due to ongoing analyses of samples from the previous patient(s). Heparinised blood and serum were obtained from patients (n = 20). Blood was drawn 24 h, 3 d, 7 d, 14 d, and 28 d after diagnosis. Each blood withdrawal was approved by the physician in charge. Missing values were caused by insufficient blood samples to perform all analyses or missing blood samples due to patient health or death or logistic issues. As control, blood was collected from non-hospitalised volunteers that were recruited from the regional community and did not fulfil the exclusion criteria (n = 20). Of note, age-related comorbidities such as hypertension were not excluded. Control subjects were exactly matched for age (±1 y) and sex but not for BMI, lifestyle factors, or comorbidities.

### Cell isolation

Peripheral blood mononuclear cells (PBMCs) were prepared from heparinised whole blood using Ficoll density gradient centrifugation followed by erythrocyte lysis as described previously^38^ and analysed immediately. Serum was obtained from clotted whole blood after centrifugation at 2000 g for 10 min. Aliquots were stored at −20 °C for further analysis. All cells from controls and patients were treated identically.

NK cells were isolated using the “MACSxpress Whole blood isolation kit” (Miltenyi Biotec) and AutoMACS according the manufacturer's instruction.

### PBMC cell culture and stimulation

PBMC were cultured in VLE RPMI 1640 medium (supplemented with stable glutamine; PAN-Biotech, Aidenbach, Germany) supplemented with 100 μg/mL streptomycin, 100 U/mL penicillin (Gibco Life Technologies, Grand Island, New York), and 4% (v/v) autologous serum or foetal calf serum (FCS; Biochrom) as indicated. PBMC were set up in triplicates (0.4 × 10^6^ cells/well in 96-well F-bottom plates) at a total volume of 200 μL/well and incubated at 37 °C and 5% CO_2_ in a humidified atmosphere. Where indicated, 4 μM SB431542 (Tocris, Bristol, UK) was added after 30 min of incubation. 20 min later 5 ng/mL human recombinant IL-15 (PeproTech, Cranbury, New Jersey), 1 ng/mL human recombinant IL-12 (Biolegend, San Diego, CA), 5 ng/mL human recombinant IL-18 (MBL, Illinois, USA), 20 μM Compound C (BioGems, CA, USA), or Dimethylformamide (DMF; Roth, Karlsruhe, Germany), were added as indicated. The indicated concentration of the compounds was selected according to a priori titration experiments and had no toxic effect. After 20 min, the cells were stimulated with heat-inactivated *S. aureus* (10^6^ bacteria/ml corresponding to one bacterium per 2 leukocytes, Invivogen, San Diego, CA), heat-inactivated *E. coli* (Invivogen, 10^7^ bacteria/ml corresponding to 5 bacteria per leucocyte), or heat-inactivated *C. albicans* (Invivogen, 10^6^ *Candida* cells/ml corresponding to one *Candida* cell per 2 leukocytes) as indicated for 18 h.

### Preparation of conditioned media and NK cell culture

For preparation of conditioned media, PBMC from healthy volunteers were stimulated with heat-inactivated *S. aureus* (0.5 × 10^6^ bacteria/ml corresponding to one bacterium per 4 leukocytes) in culture medium supplemented with 2% FCS. After 18 h, cell-free supernatants were harvested and pooled. Aliquots were stored at −20 °C until use.

Isolated NK cells (2 × 10^4^ cells/well in a total volume of 200 μL/well in 96 well U-bottom plates) were cultured in conditioned media diluted in culture medium (1:8 v/v) and supplemented with 5% FCS. 20 μM Compound C or the corresponding volume of DMF as solvent control was added as indicated. 18 h later the cells were harvested.

### Flow cytometry

For surface staining cells were incubated with antibodies against CD3 (clone OKT-3, FITC-labelled, ImmunoTools, Friesoythe, Germany), CD56 (clone CMSSB, APC-labelled, Thermo Fisher Scientific, Waltham, MA), IL12Rβ2 (clone REA333, PE-labelled, Miltenyi Biotec), and CD71 (clone CY1G4, PE/Cy7-labelled, Biolegend Inc. San Diego, USA) or with antibodies against CD3 (clone UCHT1, APC A700-labelled, BioLegend), CD56, CD98 (clone REA387, PE-labelled, Miltenyi Biotec), CD71, and CD36 (clone 5-271, FITC-labelled, BioLegend) for 12 min at 4 °C. Cells were washed with Cell Wash (BD Bioscience, Franklin Lakes, USA) before analysis.

For intracellular staining of IFN-γ, GolgiStop (0.1 μL/well, BD Biosciences) was added at the end of the 18 h incubation period. After 5 h, cells from a triplicate culture were pooled and stained with antibodies against CD3 (FITC-labelled) and CD56 (APC-labelled) as described above. Permeabilization was performed with CytoPerm/Fix (BD Bioscience) according to the manufacturer's instructions followed by staining against IFN-γ (clone 4S.B3, PE-labelled, BioLegend) for 20 min. After washing with permeabilization buffer, cells were resuspended in CellWash.

Intracellular staining of mTOR (clone MRRBY, PE-labelled, Thermo Fisher Scientific), RPS6 (clone A17020B, BV421-labelled, BioLegend Inc), or GLUT1 (clone EPR3915, APC A700-labelled, Abcam Inc, Cambridge, UK) was performed using the “FoxP3/Transcription Factor Staining Buffer Set” (Thermo Fisher Scientific) after surface staining with antibodies against CD3 (FITC-labelled) and CD56 (APC-labelled). For all analyses, corresponding isotype control stainings were performed to determine the threshold for positive staining. Zombie-K0525 (Biolegend) was used to discriminate live/dead cells (Supplementary Fig. S1). Phenotyping of NK cells was performed as described in Supplementary Material. All antibodies used were commercially available and have not been additionally validated (Supplementary Tables S1 and S2).

Data were acquired using a Cytoflex Flow cytometer (Beckman Coulter, Krefeld, Germany). Data analysis was performed using the Cytexpert 3.4 software (Beckmann Coulter) and FCS Express Flow 7 (DeNovo Software, Pasadena, USA).

Due to insufficient numbers of isolated PBMCs and missing blood samples (see above), it was not possible to perform all stainings for each patient at each time point.

### Quantification of serum cytokines

Quantification of IL-6 and GDF-15 was performed using a highly sensitive ELISA, following the manufacturer's instructions (DuoSet, R&D Systems, Biotechne). The detection limit was 0.8 pg/mL for GDF-15 and 9.4 pg/mL for IL-6.

### Statistical analysis

Since the majority of datasets deviated from normality (tested using Shapiro–Wilk test), non-parametric tests were used throughout. Graphs show median/interquartile range of individual values. For statistical evaluation, non-parametric tests such as Mann Whitney U-test or Wilcoxon signed rank test (for comparison of two data sets) and Friedman or Kruskal–Wallis test followed by Dunn's post test (for multiple comparison of more than two data sets) were used as indicated. Spearman r was used for correlation analyses between two parameters. GraphPad Prism 9 was used for statistics and the preparation of graphs.

### Role of funders

The funding organisation did not take part in the study including the experiment design, data collection, analysis, interpretation of results, or the preparation, revision and decision to submit the manuscript for publication.

## Results

### Profound and long-lasting deterioration of NK cell function during sepsis

In total, 20 patients who fulfilled the Sepsis-3 criteria (1) and 20 sex- and age-matched controls were enrolled (patient characteristics are listed in Table 1). None of the patients suffered from chronic debilitation before admission. Sepsis-inducing pathogens were *E. coli*, *S. aureus,* other Gram-negative bacteria, and *Candida* spp. (Supplementary Table S3). As expected, septic patients showed increased levels of the inflammatory markers IL-6 (p < 0.01), C-reactive protein (CRP; p < 0.001), procalcitonin (PCT; p < 0.001), lactate (p < 0.01), and lactate dehydrogenase (LDH; p < 0.01) (Supplementary Fig. S2A). In line with a large body of data, lymphopenia (Supplementary Fig. S2B; p < 0.05) and protracted reduction of HLA-DR expression on monocytes (Supplementary Fig. S2C; p < 0.001) indicated immunosuppression in septic patients. Consistently, HLA-DR expression on monocytes inversely correlated with the “sequential organ failure assessment” (SOFA) score, a measure of disease severity (Supplementary Fig. S2D; p < 0.001).

**Table 1:** Patient characteristics.

Parameter	Patients (n = 20)	Controls (n = 20)
Age,y, median (Range)	55 (21–87)	55 (22–88)
BMI, kg/m^2^, median (Range)	27.2 (22.4–41.2)	NA
Sex, n (%)		
Male	13 (65%)	13 (65%)
Female	7 (35%)	7 (35%)
SOFA Score at admission, median (IQR)	12.5 (11–15)	NA
max. SOFA Score (28-day investigation period), median (IQR)	15 (12–17)	NA
Septic schock (Sepsis-3 Criteria), n (%)	16 (80%)	NA
Catecholamine therapy, n (%)	18 (90%)	NA
Glucocorticoid therapy, n (%)	15 (75%)	NA
mechanical ventilation duration, h, median (IQR)	201 (106–305.5)	NA
Length of stay in ICU, d, median (IQR)	13 (8–18)	NA
28-day mortality, n (%)	11 (55%)	NA
Death after sepsis diagnosis, d, median (IQR)	7 (4.5–13.5)	NA
Comorbidity,n (%)		
Arterial Hypertension	4 (20%)	NA
Atrial fibrillation	1 (5%)	NA
Coronary heart disease	1 (5%)	NA
Chronic pancreatitis	2 (10%)	NA
Liver cirrhosis	2 (10%)	NA
Alcohol abuse	4 (20%)	NA
Splenomegaly	1 (5%)	NA
Microcytic, hypochromic anaemia	1 (5%)	NA
Diabetes mellitus Typ II	3 (15%)	NA
Hypothyroidism	1 (5%)	NA
Asthma bronchiale	1 (5%)	NA
COPD	1 (5%)	NA
PAD	1 (5%)	NA
Type of Infection, n (%)		
Community acquired	9 (45%)	NA
Nosocomial	11 (55%)	NA
Site of sepsis inducing infection, n (%)		
Pulmonary, respiratory	12 (60%)	NA
Gastric	3 (15%)	NA
Urinary	2 (10%)	NA
Other	3 (15%)	NA
Microbiologicially documented, n (%)		
Gram pos.	10 (50%)	NA
Gram neg.	7 (35%)	NA
Fungi	3 (15%)	NA
Positive blood culture, n (%)	12 (60%)	NA
Secondary nosocomial infection after sepsis diagnosis, n (%)	6 (30%)	NA
Diagnosis after sepsis diagnosis, d, median (IQR)	6 (5–7.75)	NA
Site of secondary infection, n (%)		
Pulmonary, respiratory	4 (67%)	NA
Gastric	1 (17%)	NA
Unknown	1 (17%)	NA
Microbiologicially documented pathogens after sepsis diagnosis, n (%)		
Gram neg.	2 (33%)	NA
Fungi	3 (50%)	NA
Viral	1 (17%)	NA
Positive blood culture, n (%)	1 (17%)	NA

BMI, Body mass index; COPD, chronic obstructive airways disease; ICU, intensive care unit; IQR, interquartile range, NA, not applicable; SOFA, sequential organ failure assessement; PAD, periphery arterial disease.

Among all patients, 30% developed secondary infections on (median) day 6 after sepsis diagnosis (Table 1). The lung was the main focus of secondary infection (Table 1). In each case of “secondary infection”, the underlying pathogen differed from the origin of the primary, sepsis-inducing infection (pathogens are listed in Supplementary Table S3). The 30-day mortality rate was 55% (Table 1), higher than in other sepsis cohorts, likely due to the high proportion of patients with septic shock and an overall high disease severity (as assessed by the SOFA score).

**Fig. 1:**
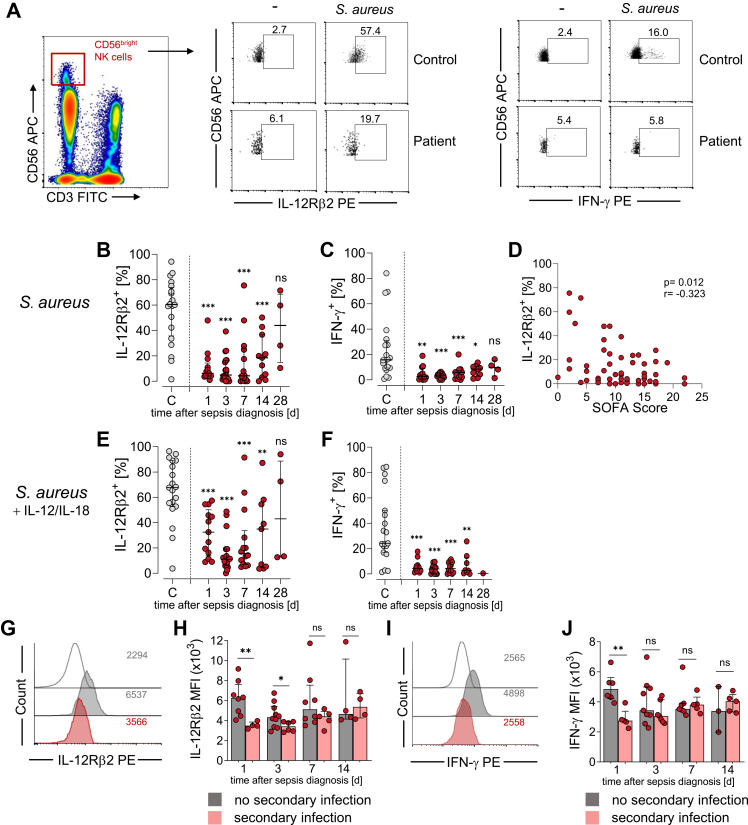
**The IL-12R/IFN-**γ **axis of CD56^bright^ NK cells is disturbed during sepsis and correlates with disease severity.** PBMCs from controls (C; n = 20) and from patients on day 1, 3, 7, 14, and 28 (n = 4–19) after diagnosis of sepsis were stimulated with heat-inactivated *S. aureus* in the presence of autologous serum. The expression of IL-12Rβ2 and IFN-γ was determined by flow cytometry. (**A**) Gating strategy for CD3^−^CD56^bright^ NK cells and representative dot plots for the expression of IL-12Rβ2 and IFN-γ by CD56^bright^ NK cells. Numbers indicate the percentage of positive cells. (**B, C)** Cumulative data of the frequency of IL-12Rβ2^+^**(B)** and IFN-γ^+^**(C)** cells among gated CD56^bright^ NK cells. (**D**) Spearman correlation of IL-12Rβ2 expression on stimulated CD56^bright^ NK cells with the SOFA Score. (**E-J)** IL-12 and IL-18 were added during stimulation of PBMC with *S. aureus*. Cumulative data of the frequency of IL-12Rβ2^+^**(E)** and IFN-γ^+^**(F)** cells among gated CD56^bright^ NK cells. **(G**–**J)** Comparison of the expression of IL-12Rβ2 and IFN-γ by CD56^bright^ NK cells from patients in terms of development of secondary infection during the observation period. Representative histograms for IL-12Rβ2 **(G)** and IFN-γ **(I).** Isotype control staining is shown in grey. Numbers indicate the median fluorescence intensity (MFI). **(H, J)** Cumulative data of the expression of IL-12Rβ2 **(H)** and IFN-γ **(J)**. Horizontal lines in the bar graphs and scatter plots indicate the median/interquartile range from individual data points. Statistical differences were tested using the Mann–Whitney test (controls vs. patients or secondary infection vs. no secondary infection (∗p < 0.05, ∗∗p < 0.01, ∗∗∗p < 0.001). Ns, not significant; SOFA, sequential organ failure assessment score.

The frequency of CD56^bright^ NK cells was reduced in septic patients on day 3 after diagnosis of sepsis (p < 0.01) and remained decreased throughout the observation period (Supplementary Fig. S3). We first examined the responsiveness of NK cells within circulating leukocytes from septic patients to a putative secondary infectious insult with *S. aureus*. NK cells themselves do not respond to *S. aureus* but require the interaction with accessory cells that provide stimulatory cytokines such as IL-12 and IL-18.^11^^,^^39^^,^^40^ Therefore, PBMCs, rather than isolated NK cells, from septic patients or control subjects were stimulated with inactivated *S. aureus* in the presence of autologous serum and the expression of IL-12Rβ2 and production of IFN-γ by CD56^bright^ NK cells were analysed in the mixed culture (gating strategy is shown in Fig. 1A).

CD56^bright^ NK cells from controls expressed the IL-12Rβ2 chain and produced IFN-γ (Fig. 1A, B, C) and TNF-α (Supplementary Fig. S4) upon exposure to *S. aureus*. In contrast, NK cells of patients from day 1 to at least day 14 after sepsis diagnosis were impaired in IL-12Rβ2 expression (p < 0.001) (Fig. 1A and B) and IFN-γ production (p < 0.01) (Fig. 1A and C). Moreover, CD56^bright^ NK cells from septic patients were impaired in TNF-α production (p < 0.05) (Supplementary Fig. S4). The IL-12Rβ2 expression negatively correlated with disease severity (SOFA score) (p < 0.05) (Fig. 1D).

A reduced cytotoxicity of NK cells from septic patients against tumour cells has been reported.^41^ In contrast, we observed that the CD16-or NKp30-mediated degranulation of NK cells from septic patients was not affected (p > 0.05) (Supplementary Fig. S5). CD56^bright^ NK cells from septic patients displayed an activated phenotype and expressed enhanced levels of the exhaustion markers PD-1 (p < 0.05), CTLA-4 (p < 0.01), and TIGIT (p < 0.05) whereas the expression of activating receptors such as NKG2D was unchanged (p > 0.05) (Supplementary Fig. S6).

Monocytes require additional stimulation with IFN-γ to release IL-12 in response to bacterial components.^42^ Monocytes from septic patients are impaired in the secretion of IL-12 upon stimulation with the combination of *S. aureus* and recombinant IFN-γ.^43^ To address whether an insufficient secretion of cytokines by accessory cells was responsible for the reduced activity of NK cells during sepsis, the cultures were supplemented with recombinant IL-12 and IL-18. Despite excess IL-12 and IL-18, NK cells from septic patients maintained impaired IL-12Rβ2 chain expression (p < 0.001) (Fig. 1E) and IFN-γ production (p < 0.001) (Fig. 1F). Of note, on day 1 after sepsis diagnosis, NK cells from patients who later developed a secondary infection expressed lower levels of the IL-12Rβ2 chain (p < 0.01) (Fig. 1G and H) and IFN-γ (p < 0.01) (Fig. 1I and J) whereas no difference was observed between survivors and non-survivors (Supplementary Fig. S7). Thus, the IL-12/IFN-γ axis of NK cells is severely compromised during sepsis and is associated with disease severity and susceptibility to secondary infections.

### Circulating growth/differentiation factor 15 correlates with disease severity but is not the main driver of NK cell dysfunction during sepsis

**Fig. 2:**
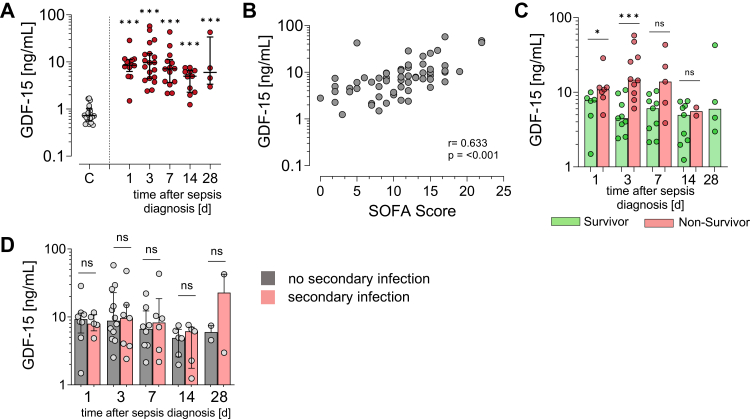
**High levels of GDF-15 circulate during sepsis and correlate with disease severity.** GDF-15 in the sera from controls (C; n = 20) and from patients on day 1, 3, 7, 14, and 28 after sepsis diagnosis (n = 4–19) was quantified. **(A)** Concentration of GDF-15. Horizontal lines indicate the median/interquartile range. **(B)** Spearman correlation of GDF-15 in septic patients with the SOFA Score. **(C)** GDF-15 in survivors and non-survivors. Bars indicate the median value. **(D)** Comparison of GDF-15 in patients according to development of secondary infection. Data show the median/interquartile range of individual values. Statistically significant differences between controls and patients **(A),** survivors and non-survivors **(C),** and development of secondary infection **(D)** were tested using the Mann–Whitney Test. ∗, p < 0.05; ∗∗∗p < 0.001. GDF-15, growth/differentiation factor 15; ns, not significant.

NK cell function is dictated by environmental signals and by cell–intrinsic pathways. Therefore, we asked whether suppressive factors in the serum were responsible for the impaired IL-12/IFN-γ response of NK cells during sepsis. We previously identified growth/differentiation factor (GDF) 15, a member of the TGF-β family, in the serum of severely injured patients as a potent inhibitor of NK cell function.^38^ In line with this, septic patients exhibited markedly increased serum levels of GDF-15 throughout the observation period from day 1 until day 28 after diagnosis (p < 0.001) (Fig. 2A) that correlated with disease severity (p < 0.001) (Fig. 2B). Significantly higher concentrations of GDF-15 circulated in non-survivors than in survivors at 1 d (p < 0.05) and 3 d (p < 0.001) after sepsis diagnosis (Fig. 2C). Levels of circulating GDF-15 did not differ between patients who developed secondary infection and those who did not (Fig. 2D).

**Fig. 3:**
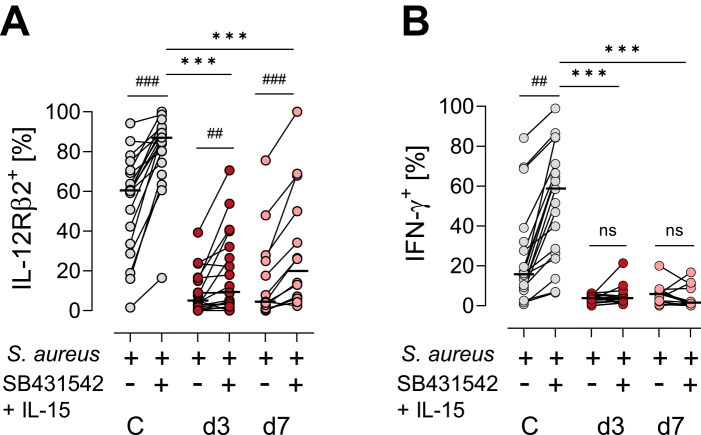
**Targeting the TGF-βRI does not restore NK cell function during sepsis.** PBMC from control subjects (C; n = 19) and from patients at day 3 and 7 after sepsis diagnosis (d3, d7; n = 13–19) were stimulated with inactivated *S. aureus* in the presence of autologous serum, with or without SB431542 (an inhibitor of the TFG-βRI) and IL-15. Expression of IL-12Rβ2 **(A)** and IFN-γ **(B).** The values for cells stimulated with *S. aureus* alone are the same as shown in Fig. 1B and C. Horizontal lines indicate the median/interquartile range from individual data points. Statistically significant differences between culture conditions were tested using the Wilcoxon-signed rank test (##, p < 0.01; ###, p < 0.001). Differences between controls and patients were tested using the Kruskal–Wallis test followed by Dunn's multiple comparison test (∗∗∗; p < 0.001). TFG-βRI, Transforming growth factor receptor I.

Our previous work showed that circulating GDF-15 signals through the TGF-βRI (ALK5)/Smad 1/5/8 pathway^38^ and that inhibition of the TGF-βRI improves the function of NK cells from severely injured patients.^37^ This TGF-βRI-mediated mechanism might also contribute to NK cell dysfunction during sepsis documented in Fig. 1. To address this, SB431542, a small molecule inhibitor of the TGF-βRI, and IL-15 that synergises with SB431542^37^ were added before stimulation of PBMCs with *S. aureus*. Inhibition of the TGF-βRI in combination with IL-15 slightly increased the expression of the IL-12Rβ2 on the patients’ NK cells (p < 0.01) but by far did not reach the levels from controls (p < 0.001) (Fig. 3A). The combination of SB431542 and IL-15 strongly increased the release of IFN-γ by NK cells from controls (p < 0.001) but was ineffective on NK cells from septic patients (p > 0.05) (Fig. 3B). Likewise, GW788388, an alternative inhibitor of the TGF-βRI, in combination with IL-15 failed to improve NK cell function (p > 0.05) (Supplementary Fig. S8A and B).

To test the assumption that a yet unknown mediator in the serum was responsible for the suppression of NK cells during sepsis, cultures were set up in the absence of autologous serum. The patients’ NK cells were still inferior to cells from controls in the expression of IL-12Rβ2 (p < 0.01) and IFN-γ (p < 0.05) (Supplementary Fig. S9A). The absence of autologous serum during stimulation of NK cells from septic patients did not significantly improve the expression of IL-12Rβ2 and IFN-γ by NK cells throughout the observation period (Supplementary Fig. S9B).

Thus, high levels of circulating GDF-15 in septic patients are associated with morbidity but GDF-15 was not the main driver of NK cell suppression.

### Reduced nutrient sensing in NK cells during sepsis

**Fig. 4:**
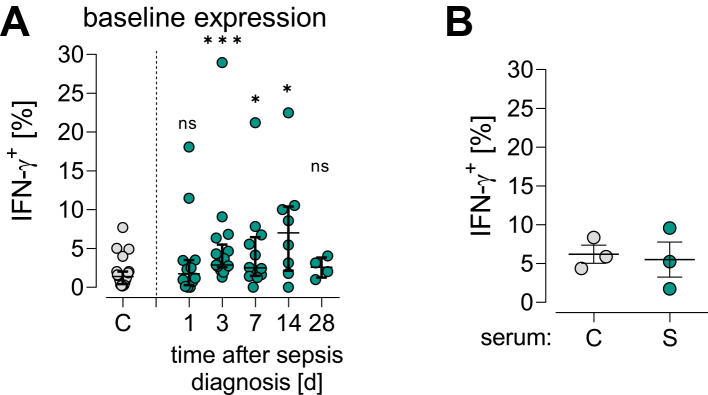
**NK cells transiently produce IFN-**γ **at baseline during sepsis. (A)** PBMC from control subjects (C; n = 20) and from patients on day 1, 3, 7, 14, and 28 after diagnosis; (n = 4–18) were cultured in medium supplemented with autologous serum (termed baseline). **(B)** PBMC from controls (n = 3) were cultured in medium supplemented with pooled sera from controls (C) or from septic patients (S) obtained 1 d after sepsis diagnosis. The frequency of IFN-γ^+^ cells among CD56^bright^ NK cells was determined. Horizontal lines indicate the median/interquartile range. Statistical differences between samples from controls and patients were tested using the Mann–Whitney test (∗p < 0.05, ∗∗∗p < 0.001).

The previous data suggested that the disturbed IL-12R/IFN-γ axis of NK cells from septic patients was not primarily caused by a suppressive milieu in the circulation. Therefore, we asked whether NK cells underwent cell-intrinsic changes early during sepsis that might later affect their responsiveness to opportunistic pathogens. NK cells produced low baseline levels of IFN-γ (without stimulation with *S. aureus*; Fig. 4A) at 3 d after sepsis diagnosis (p < 0.001), indicating that NK cells had received unknown stimulatory signals at an earlier time point *in vivo*. Diverse pathogen- or tissue damage-associated molecules circulate during sepsis and might stimulate NK cells which we tested by exposure of NK cells from donors to the serum of septic patients. However, there was no evidence for a patients’ serum-induced increase in IFN-γ secretion (Fig. 4B).

**Fig. 5:**
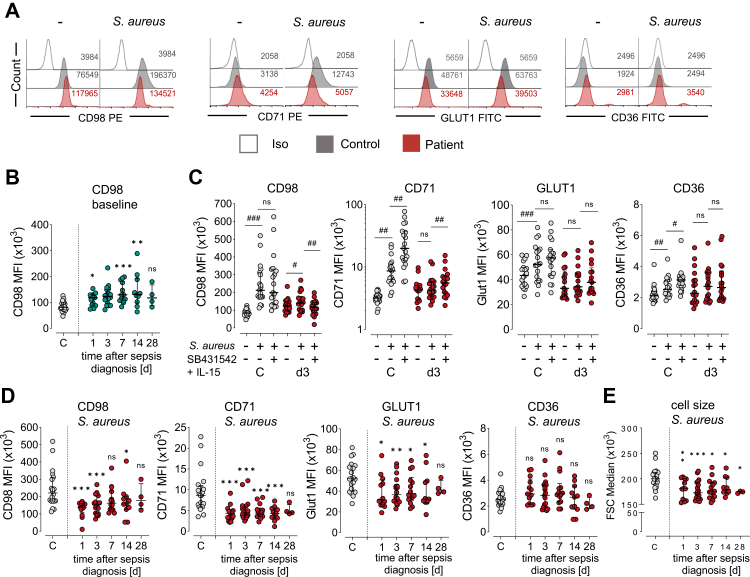
**Nutrient sensing by NK cells is disturbed during sepsis.** PBMCs from controls (C; n = 20) and from patients on day 1, 3, 7, 14, and 28 (n = 4–19) after diagnosis of sepsis were cultured in medium (termed baseline) **(A, B)** or stimulated with heat-inactivated *S. aureus*, SB431542 (an inhibitor of the TFG-βRI), and IL-15 as indicated (each in the presence of autologous serum) **(C**–**E)**. The expression of nutrient receptors and transporters were determined by flow cytometry. **(A)** Representative histograms for the expression of CD98 (amino acid transporter), CD71 (transferrin receptor 1), GLUT1 (glucose transporter), CD36 (fatty acid transporter) of gated CD56^bright^ NK cells from control and septic patients. Numbers indicate the median fluorescence intensity (MFI). **(B)** Baseline expression of CD98 on CD56^bright^ NK cells from controls and septic patients. **(C)** Impact of *S. aureus* and SB431542/IL-15 on the expression of nutrient transporters on CD56^bright^ NK cells from controls (n = 19) and septic patients on day 3 after diagnosis (n = 18). **(D)** Kinetics of the expression of nutrient transporters on CD56^bright^ NK cells upon stimulation with *S. aureus*. **(E)** Cell size represented by the forward scatter (FSC) of CD56^bright^ NK cells from controls and septic patients upon stimulation with *S. aureus*. Horizontal lines indicate the median/interquartile range from individual values. Statistically significant differences were tested using Mann–Whitney test (**B**, **D**, **E**; controls vs. patients; ∗p < 0.05; ∗∗p < 0.01; ∗∗∗p < 0.001) or Friedman test followed by Dunn's multiple comparison test (**C**; differences between culture conditions; ^#^p < 0.05; ^##^p < 0.01; ^###^p < 0.001). Iso, isotype control.

The function of NK cells is intrinsically regulated by their cellular metabolism.^20^ Accordingly, sensing and uptake of nutrients are relevant for NK cell function and might be modulated early during sepsis. We evaluated the expression of diverse nutrient transporters/receptors at baseline (without stimulation with *S. aureus*) and observed a prominent protracted constitutive expression of the amino acid transporter CD98 on NK cells early after sepsis diagnosis (p < 0.001) which further indicates prior activation of the cells *in vivo* (Fig. 5 A, B). CD98 levels correlated with the elevated IFN-γ production at baseline (p < 0.001) (Supplementary Fig. S10A). The patients’ serum did not induce the expression of CD98 on NK cells from donors (Supplementary Fig. S10B).

The baseline expression (without stimulation with *S. aureus*) of the transferrin receptor CD71 and the fatty acid transporter CD36 on NK cells transiently increased on day 3 (p < 0.05) and day 7 (p < 0.001), respectively, after sepsis diagnosis, whereas the glucose transporter GLUT1 did not change significantly during the observation period (Supplementary Fig. S11).

In comparison to CD56^bright^ NK cells, CD56^dim^ NK cells from septic patients expressed enhanced levels of CD98 (p < 0.01) and CD71 (p < 0.05) at baseline though delayed and to a lesser extent, respectively, but not IFN-γ (Supplementary Fig. S12B–D). In contrast, the expression of GLUT1 was decreased on CD56^dim^ NK cells (p < 0.05) (Supplementary Fig. S12E).

Upon activation, NK cells enhance the expression of nutrient transporters and receptors, enabling them to cope with the increased demand for biomolecules. As expected, NK cells from controls displayed enhanced expression of either nutrient transporter/receptor when exposed to *S. aureus* (p < 0.01) (Fig. 5C). Addition of the TGF-βRI inhibitor SB431542 and IL-15 further boosted the level of CD71 (p < 0.01) but had only minor or no effects on CD36 (p < 0.05), CD98 (p > 0.05), and GLUT1 (p > 0.05) (Fig. 5C). Although NK cells from septic patients displayed slightly increased baseline levels of CD98 and CD71 (Fig. 5B and Supplementary Fig. S11), their expression of the nutrient transporters/receptor increased only weakly, if at all, upon stimulation with *S. aureus* (Fig. 5C). Consequently, the expression of CD98 (p < 0.001), CD71 (p < 0.001), and GLUT1 (p < 0.01) in the presence of *S. aureus* remained clearly reduced in comparison to NK cells from controls for at least 14 d (Fig. 5D). The expression of CD71 on NK cells from septic patients only slightly, if at all, increased in the presence of the TGF-βRI inhibitors and IL-15 (p < 0.01) but by far did not reach the levels observed on NK cells from controls (Fig. 5C and Supplementary Fig. S8C). The expression of nutrient transporters on CD56^bright^ NK cells upon stimulation with *S. aureus* did not differ significantly with respect to development of secondary infection (Supplementary Fig. S13A–D) or survival (Supplementary Fig. S14A–D).

In line with previous reports,^22^ CD56^dim^ NK cells from control subjects expressed reduced levels of IL-12Rβ2 (p < 0.001) and IFN-γ (p < 0.001) after exposure to *S. aureus* in comparison to CD56^bright^ NK cells (Supplementary Fig. S15A and B). The expression of IL-12Rβ2 (p < 0.001) and IFN-γ (p < 0.05) by CD56^dim^ NK cells from septic patients but not the expression of CD71 was reduced in comparison to cells from control subjects (Supplementary Fig. S15C–H).

Increased anabolic activity of stimulated NK cells is reflected by an enlarged cell size.^23^ In line with the impaired nutrient sensing after stimulation with *S. aureus*, CD56^bright^ NK cells from septic patients were smaller than cells from controls throughout the observation period (p < 0.01) (Fig. 5E). Thus, the profound suppression of the IL-12/IFN-γ axis in CD56^bright^ NK cells during sepsis is associated with a decline in nutrient sensing, compromised nutrient uptake, and anabolic processes.

### Inhibition of AMPK counteracts impaired mTORC1 activity and IFN-γ production during sepsis

Considering that mTORC1 is the central hub in cell metabolism^23^ we evaluated whether the impaired nutrient sensing and reduced cell size of NK cells during sepsis were associated with a disturbed regulation of mTORC1.

**Fig. 6:**
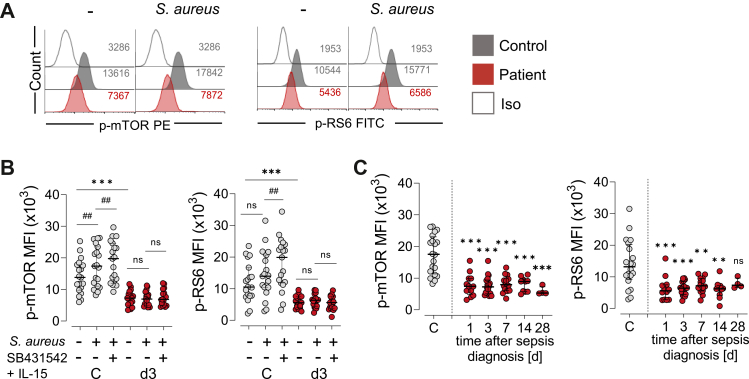
**Persistent disturbance of mTORC1 activation in NK cells during sepsis.** PBMCs from controls (C; n = 20) and from patients on day 1, 3, 7, 14, and 28 (n = 4–19) after diagnosis of sepsis were stimulated with heat-inactivated *S. aureus*, SB431542 (an inhibitor of the TFG-βRI), and IL-15 as indicated (each in the presence of autologous serum). The phosphorylation of mTOR and RS6 in CD56^bright^ NK cells was determined. **(A)** Representative histograms for the phosphorylation of mTOR and RS6 in gated CD56^bright^ NK cells from control and septic patients. Numbers indicate the median fluorescence intensity (MFI). **(B)** Impact of *S. aureus* and SB431542/IL-15 on the phosphorylation of mTOR and RS6 in CD56^bright^ NK cells from controls (n = 19) and septic patients on day 3 after diagnosis (n = 18). **(C)** Activation of mTOR and RS6 in CD56^bright^ NK cells upon stimulation with *S. aureus* during the course of sepsis. Horizontal lines indicate the median/interquartile range from individual values. Statistically significant differences were tested using Friedman test followed by Dunn's multiple comparison test (**B**; differences between culture conditions; ^##^p < 0.01) or Mann–Whitney test (**C**; controls vs. patients; ∗∗p < 0.01; ∗∗∗p < 0.001). Iso, isotype control.

In comparison with controls, NK cells from septic patients showed reduced baseline phosphorylation of mTOR (p < 0.001) and its downstream target RS6 (p < 0.001), a readout of mTORC1 activity (Fig. 6A and B). In NK cells from controls, the activity of mTORC1 (p < 0.01) increased after exposure to *S. aureus* and moreover in the presence of the TGF-βRI inhibitor SB431542 in combination with IL-15 (p < 0.01) as expected^25^^,^^26^ (Fig. 6A and B). In contrast, stimulation with *S. aureus*, alone or in combination with SB431542 and IL-15, failed to activate mTORC1 in NK cells from septic patients (Fig. 6A and B). The suppressed activation of mTORC1 in the patients’ NK cells was maintained throughout the entire observation period (p < 0.01) (Fig. 6C) and was associated with the development of secondary infection (on day 3 after sepsis diagnosis (p < 0.05); Supplementary Fig. S13E and F) but not with survival (Supplementary Fig. S14E and F). CD56^dim^ NK cells from septic patients likewise displayed reduced activation of mTORC1 (p < 0.001) (Supplementary Fig. S15I and K).

The deterioration of the IL-12R/IFN-γ axis and the impaired activation of mTORC1 in NK cells during sepsis was also observed upon stimulation with *C. albicans* (each p < 0.05) and, in part, with *E. coli* (IL-12Rβ2, p < 0.05) (Supplementary Fig. S16).

**Fig. 7:**
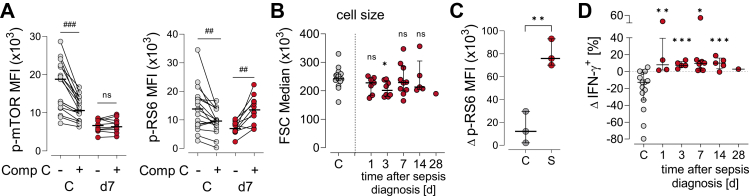
**Inhibition of AMPK improves mTORC1 activity and functionality of NK cells during sepsis. (A, B, D)** PBMCs from controls (C; n = 20) and from patients on day 1, 3, 7, 14, and 28 (n = 4–19) after diagnosis of sepsis were stimulated with heat-inactivated *S. aureus* in the presence of Compound C (an AMPK inhibitor; +) or DMF as solvent control (−) each in autologous serum. **(A)** Phosphorylation of mTOR and RS6 in CD56^bright^ NK cells was determined. **(B)** Cell size of CD56^bright^ NK cells PBMC in the presence of Compound C. **(C)** Purified NK cells from controls (C; n = 3) and from patients (S; day 7 after diagnosis; n = 3) were cultured in conditioned media of PBMC from healthy donors stimulated with inactivated *S. aureus* each in the presence or absence of Compound C. The change of phosphorylated RS6 (Δp-RS6; MFI p-RS6 in Compound C – MFI p-RS6 in DMF) in gated CD56^bright^ NK cells was determined. **(D)** Differences in IFN-γ expression of CD56^bright^ NK cells after treatment of PBMC with Compound C (ΔIFN-γ; %IFN-γ^+^ in Compound C – %IFN-γ^+^ in DMF). Horizontal lines indicate the median/interquartile range of individual values. Statistically significant differences were tested using the Mann–Whitney test (controls vs. patients; ∗p < 0.05; ∗∗p < 0.01; ∗∗∗p < 0.001) or Wilcoxon signed-rank test (differences between culture conditions; ^#^^#^p < 0.01; ^##^^#^p < 0.001). DMF, Dimethylformamide; Comp C, Compound C.

mTORC1 is negatively regulated by AMPK.^28^ Therefore, we examined whether Compound C, an inhibitor of AMPK, could restore the activation of mTORC1 in NK cells from septic patients. Compound C reduced the activation of mTOR and RS6 in NK cells from controls (p < 0.01) (Fig. 7A). In contrast, Compound C increased the activation of RS6 (p < 0.01) without effecting mTOR activation in NK cells from septic patients (Fig. 7A). In line with the increased activation of mTORC1 in the presence of Compound C, the cell size of NK cells from septic patients became equivalent to the cell size from controls (Fig. 7B).

We asked whether the increased RS6 activity in NK cells from patients was a direct effect of Compound C on the cells or secondary to the impact of the substance on other cell types within PBMC. Since isolated NK cells do not respond to *S. aureus* bacteria we had to mimic the “cytokine environment” that mediates the stimulation of NK cells within PBMC. Therefore, PBMC from healthy volunteers were stimulated with *S. aureus* and the supernatant of these cells that contained all relevant cytokines was collected as “conditioned medium”. NK cells were purified from patients and control subjects and cultured in this conditioned medium in the absence or presence of Compound C. Compound C strongly increased RS6 activation in isolated NK cells from septic patients (p < 0.01) but had a minor effect on NK cells from controls (Fig. 7C). Finally, the treatment with Compound C improved the capacity of NK cells from septic patients to release IFN-γ at each time point after diagnosis in comparison with NK cells from controls (p < 0.01) (Fig. 7D). Thus, NK cells from septic patients show a strong deficit in mTORC1 activation at baseline and upon stimulation that may be at least partially restored by inhibition of AMPK.

## Discussion

The present study comprehensively investigated longitudinal changes in NK cell function and its association with nutrient sensing during human sepsis. We provide evidence that NK cells exhibit a Janus-faced behaviour during sepsis: CD56^bright^ NK cells (but not CD56^dim^ NK cells) produced IFN-γ at baseline, a hallmark of pro-inflammatory activity, but simultaneously showed a severely impaired responsiveness to a secondary bacterial challenge reflecting an immunosuppressive phenotype. Previous studies focused either on the pro-inflammatory activity or on the immunosuppressive phenotype and therefore have led to confusion about the beneficial or detrimental role of NK cells during sepsis.^31^ A common view is that the innate immune system is largely responsible for the hyperinflammatory response whereas an inadequate function of the adaptive immune system results in the development of immunosuppression during sepsis.^44^ Our findings extend this concept and indicate that these opposing states do not only rely on different branches of the immune system but moreover co-exist within an individual cell population, such as NK cells, already very early after sepsis diagnosis.

The profound and long-lasting failure to respond to a secondary challenge with *S. aureus* was linked to a disruption of the IL-12R/IFN-γ axis in NK cells from septic patients which was associated with increased morbidity. The activation of NK cells in our model of secondary bacterial challenge depends on their cross-talk with “accessory cells” such as monocytes that provide innate cytokines like IL-12.^40^ Importantly, monocytes require additional stimulation with IFN-γ provided by NK cells to achieve their full capacity for IL-12 production.^38^^,^^42^^,^^45^ During sepsis and after major injury, monocytes release reduced levels of IL-12^43^^,^^46^ and thereby might contribute to the impaired function of NK cells that we show here. However, a reduced availability of IL-12 did not fully account for the functional reprogramming of NK cells during sepsis as the disrupted IL-12R/IFN-γ axis persisted even in the presence of excess IL-12 and IL-18. A similar disruption of the IL-12R/IFN-γ axis exists in NK cells from patients after surgical or traumatic injury.^38^^,^^47^ Of note, NK cell dysfunction after major surgery occurs despite intact IL-12 synthesis by monocytes.^47^ Altogether, these findings support the notion that functional reprogramming of NK cells develops during diverse types of severe systemic inflammation largely independent of monocyte performance.

In healthy individuals, circulating NK cells migrate to the site of infection where they release IFN-γ to support the elimination of invading pathogens.^48^ Thus, in septic patients, the disturbed IL-12R/IFN-γ axis may compromise immune defence mechanisms, particularly in tissues vulnerable to secondary infection, such as the lung. Consistent with this assumption, NK cells from patients who later developed secondary infections displayed reduced expression of IL-12R and IFN-γ in comparison to NK cells from patients who remained free of infectious complications. In murine polymicrobial sepsis, NK cells that appear in the lung during secondary pneumonia produce less IFN-γ and moreover suppress the activity of accessory cells.^49^^,^^50^ A similar behaviour of NK cells in human sepsis would likewise impair the interaction with macrophages/dendritic cells in the lung and favour the susceptibility to nosocomial pneumonia. The analyses of human lung NK cells during sepsis are challenging but are indispensable to strengthen this hypothesis in future work.

The function of NK cells may be positively and negatively regulated by the diverse signals they receive from the microenvironment. A well-known example is the tumour microenvironment that suppresses the anti-tumoral function of NK cells.^51^ We have previously shown that GDF-15 which circulates in severely injured patients is such an environmental factor. GDF-15 mediates signalling through TGF-βRI and contributes to the long-lasting dysregulation of the IL-12R/IFN-γ axis of NK cells after severe injury.^38^ In line with previous reports^52^ we observed very high levels of circulating GDF-15 especially in non-surviving septic patients throughout the observation period. Inhibition of the TGF-βRI reinforced the IL-12R/IFN-γ axis of NK cells from severely injured patients in synergy with recombinant IL-15.^37^ In contrast, the same combination of TGF-βRI inhibitor and IL-15 only marginally increased the expression of the IL-12Rβ2 and failed to improve the production of IFN-γ from NK cells during sepsis. We conclude that circulating GDF-15 is associated with morbidity and mortality in sepsis but is not primarily responsible for the deterioration of the IL-12R/IFN-γ axis in NK cells.

Given the minor impact of the microenvironment, we investigated potential cell-intrinsic mechanisms that regulated NK cell function during sepsis. The constitutive production of IFN-γ at baseline suggested prior activation of CD56^bright^ NK cells *in vivo* and correlated with increased expression of the amino acid transporter CD98. In contrast, CD56^dim^ NK cells displayed enhanced levels of nutrient transporters at baseline but there was no association with IFN-γ production. The uptake of essential amino acids through CD98/LAT1 promotes the production of IFN-γ via activation of mTORC1 in NK cells from healthy individuals.^21^^,^^22^ NK cells from septic patients did not exhibit increased mTORC1 activation suggesting that CD98 does not drive IFN-γ through this pathway. CD98 is also known to stabilise integrin activation and thereby may modulate diverse signalling pathways^53^ that contribute to the production of IFN-γ in NK cells.^54^ Whether such pathways are independent of mTORC1 and support the baseline synthesis of IFN-γ in CD56^bright^ NK cells during sepsis remains an open question. Thus, further investigation is needed to unravel the origin and significance of the constitutively increased expression of CD98 and IFN-γ by CD56^bright^ NK cells during sepsis.

Monocytes and T lymphocytes from septic patients display impaired metabolic plasticity (the switch of metabolic pathways upon activation) which causes tolerance and exhaustion, respectively, and favours the development of immunosuppression.^55^, ^56^, ^57^ The regulation of this process of metabolic rewiring during sepsis is not yet fully understood. The immunosuppressive phenotype of NK cells, as indicated by their reduced responsiveness to *S. aureus*, was associated with markedly decreased activation or expression of the key metabolic regulator mTORC1 and nutrient receptors/transporters, including CD98, CD71, and GLUT1. Additionally, NK cells from septic patients were significantly smaller in size compared to those from controls. Collectively, these findings support the hypothesis that the shift to anabolic metabolism, which is essential for NK cell activation in healthy individuals, was disturbed during sepsis–a loss of metabolic plasticity similar to that seen in monocytes and T cells. While metabolic flux analysis could help validate this hypothesis, its application has been limited by the low yield of CD56^bright^ NK cells we could isolate from septic patients.

CD56^bright^ NK cells from septic patients displayed increased expression of the immune checkpoint molecules PD-1, CTLA-4, and TIGIT which are associated with impaired NK cell function.^58^ PD-1 signalling in T cells inhibits the activation of mTORC1 and metabolic reprogramming upon cellular activation.^59^ Likewise, PD-1 signalling in CD56^bright^ NK cells of septic patients might contribute to the decreased activation of mTORC1 and production of IFN-γ, which remains to be evaluated in future studies.

As previously demonstrated, sterile inflammation in severely injured patients also leads to the inhibition of mTOR activation in NK cells.^37^ In this case, mTOR activity and the expression of IL-12Rβ2 and IFN-γ can be restored through treatment with recombinant IL-15, a well-established activator of mTORC1, particularly when combined with a TGF-βRI inhibitor.^37^ Notably, the same combination of IL-15 and TGF-βRI inhibitor completely failed to activate mTORC1 and to induce IFN-γ synthesis in NK cells from septic patients. Based on these findings, we propose that the impaired mTORC1 activity is a central mechanism underlying NK dysfunction during sepsis. Of note, the expression of IL-12Rβ2 could be restored by the combination of IL-15 and TGF-βRI as it is independent of mTORC1.^37^

The substantial inhibition of mTORC1 in NK cells implied the involvement of a potent counter-regulatory mechanism that was active during sepsis, such as regulation by AMPK. There exist only a few studies that address the relevance of AMPK in immune cells during sepsis and even less examined the balance between mTORC1 and AMPK. The majority of available data on AMPK in sepsis originate from animal studies, which suggest that AMPK activity and function varies depending on the cell type, tissue, and disease stage.^60^ Initial activation of AMPK in myeloid cells during sepsis limits hyperinflammation and enhances survival.^61^ An imbalance between mTORC1 and AMPK exists in T lymphocytes during sepsis and treatment with IL-7 partially restores mTORC1 activity and reverses T cell exhaustion.^57^

We provide evidence that treatment with an inhibitor of AMPK enhanced the activity of mTORC1 and at least slightly increased the production of IFN-γ in NK cells from septic patients. This finding supports the hypothesis that like T cells, NK cells exhibit a cell-intrinsic imbalance between mTORC1 and AMPK during sepsis. However, due to the limited number of NK cells available from septic patients, we were unable to directly quantify the activity AMPK in these cells. More detailed studies on human NK cells during sepsis are required to elucidate changes in the balance between mTORC1 and AMPK and the contribution of these key metabolic regulators to NK cell dysfunction.

Besides T cells and NK cells, also monocytes from septic patients present reduced activity of mTORC1 which contributes to the development of tolerance, a hallmark of sepsis-induced immunosuppression.^55^ Treatment with IFN-γ restores mTORC1 activity and cytokine secretion in tolerant monocytes.^55^^,^^62^ This suggests that IFN-γ could serve as a promising therapeutic approach to enhance immune defence during sepsis.^63^ However, because IFN-γ affects a broad range of biological processes in both immune and non-immune cells, its systemic application may cause severe side effects in critically ill patients.^64^ Therefore, increasing the local availability of IFN-γ at the site of infection could improve monocyte function while minimising adverse effects in other compartments. We propose that targeting NK cells to restore their IL-12R/IFN-γ axis is a promising strategy to strengthen immune defence in infected tissues where phagocytes and NK cells interact. Advances in nanoparticle-based therapies, which have already shown success in delivering therapeutics to immune cells might offer a feasible approach to enhance NK cell function *in vivo*.^65^

Several limitations of our study should be considered: the small number of patients included in this study and its design as exploratory single centre study restricts the generalisability of our findings to all patients. Patients and control subjects were not matched for BMI, lifestyle factors, or comorbidities such as type 2 diabetes or arterial hypertension. These conditions are associated with chronic low-grade systemic inflammation and may therefore independently influence NK cell metabolic adaptation and cytokine secretion, representing potential sources of residual confounding that we were unable to control for in the present study. However, we note that the direction of this confounding is unlikely to fully account for the observed findings. If such conditions were unequally distributed in favour of the patient group or more prevalent among controls, subgroups would be visible. Visual inspection of the data did not reveal apparent subgroups or clustering patterns within either the patient or control group that would suggest a dominant confounding structure driven by a specific comorbidity subpopulation. While this observation is informal and does not substitute for formal confounder adjustment, it provides modest reassurance against gross confounding. We acknowledge that definitive conclusions regarding the independence of the reported NK cell alterations from these variables will require larger studies with prospective and systematic recording of BMI, lifestyle factors, and comorbidities, enabling formal multivariable adjustment. Additionally, the low number of leukocytes obtained from patients limited the feasibility of further investigation of underlying mechanisms such as Seahorse analyses and quantification of activated AMPK. While our data revealed an association between nutrient sensing and altered NK cell function and metabolism, they do not prove a causal relationship. Future studies using genetic editing or *in vivo* interventions with AMPK agonists/inhibitors are required to further validate causality. Since Compound C inhibits multiple kinases beyond AMPK, its effect on mTORC1 in NK cells from septic patients might involve additional, yet unidentified signalling pathways. We point out that our study examined the reactivity of leukocytes to inactivated microorganisms. Unlike inactivated bacteria, living bacteria release toxins and bacterial mRNA but less cell wall components such as peptidoglycan or lipoteichoic acid. Therefore, the response of NK cells to inactivated *S. aureus* that we describe here might not fully reflect the response to viable *S. aureus* bacteria.

Finally, our experiments mainly focused on *S*. *aureus* as a model organism for nosocomial infection. First data provide evidence that sepsis also interfered with the activity of NK cells upon exposure to *C. albicans* and *E. coli* but there were pathogen-dependent differences that should be elaborated in more detail in subsequent studies.

A weakness is that all data on NK cells relied on flow cytometry. The implementation of additional experimental approaches in future studies could strengthen the reliability of our data.

In summary, we demonstrate here a profound and long-lasting impairment of the IL-12R/IFN-γ axis of NK cells during human sepsis which correlated with increased morbidity and enhanced susceptibility to secondary infections. The dysfunction of NK cells was associated with reduced nutrient sensing and suppressed activity of mTORC1, the key regulator of cellular metabolism. Overall, our findings indicate that NK cells fail to undergo metabolic reprogramming during sepsis which might contribute to their functional deficit. In the acute phase of the disease, sepsis is characterised by a profound shift toward anaerobic, catabolic metabolism with high gluconeogenesis rates, hyperglycemia, high lactate levels, and insulin resistance in varying degrees and combinations.^36^ The dysfunction of NK cells during sepsis may arise independently of these global metabolic and endocrine derangements or be part of them. Regardless of the underlying mechanisms, our findings illustrate that restoring mTORC1 activity in NK cells is an attractive therapeutic strategy to boost NK function for improved immune defence against nosocomial infections during sepsis.

## Contributors

AvdW, MG, DM, FZ, ES, CW, MC designed and performed the experiments, analysed, and interpreted the data, and wrote the manuscript; BB, BS, and IR interpreted and discussed the data, and supported in manuscript writing. SV, NK, FH, KS, MMB, TB, and MD provided material of the patients and contributed to the study design; SBF supervised the study, designed the experiments, interpreted the data, and wrote the manuscript. AvdW, CW, MC, and SBF accessed and verified the underlying data. All authors read and approved the final version of the manuscript.

## Data sharing statement

Underlying data for this study are included in the article and as Supplementary Material. There are no data that will be shared.

## Declaration of interests

The authors declare that they have no competing interests.

## Appendix A Supplementary data

Supplemental Materials and Methods

Reagents antibodies
