# Supplementary material for: Disturbed metabolic adaptation drives natural killer cell dysfunction in association with nosocomial infection during human sepsis
Source: eBioMedicine. 2026 Jun 26;129:106345. doi: 10.1016/j.ebiom.2026.106345 (PMC13324300; doi:10.1016/j.ebiom.2026.106345)
Supplement: Supplementary Figures [file mmc1.pdf]

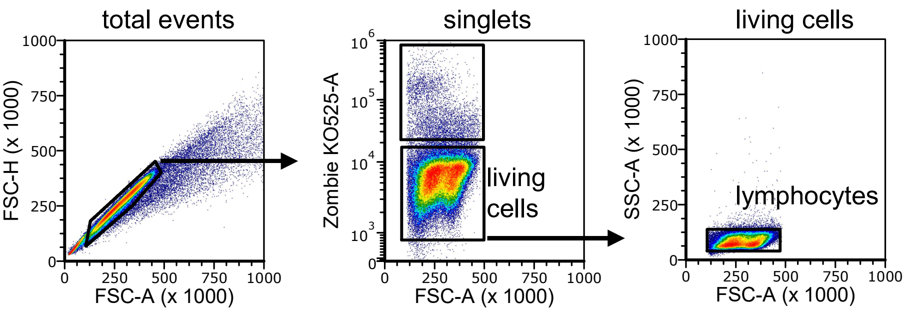

Suppl. Figure 1. Gating strategy for exclusion of dead cells.

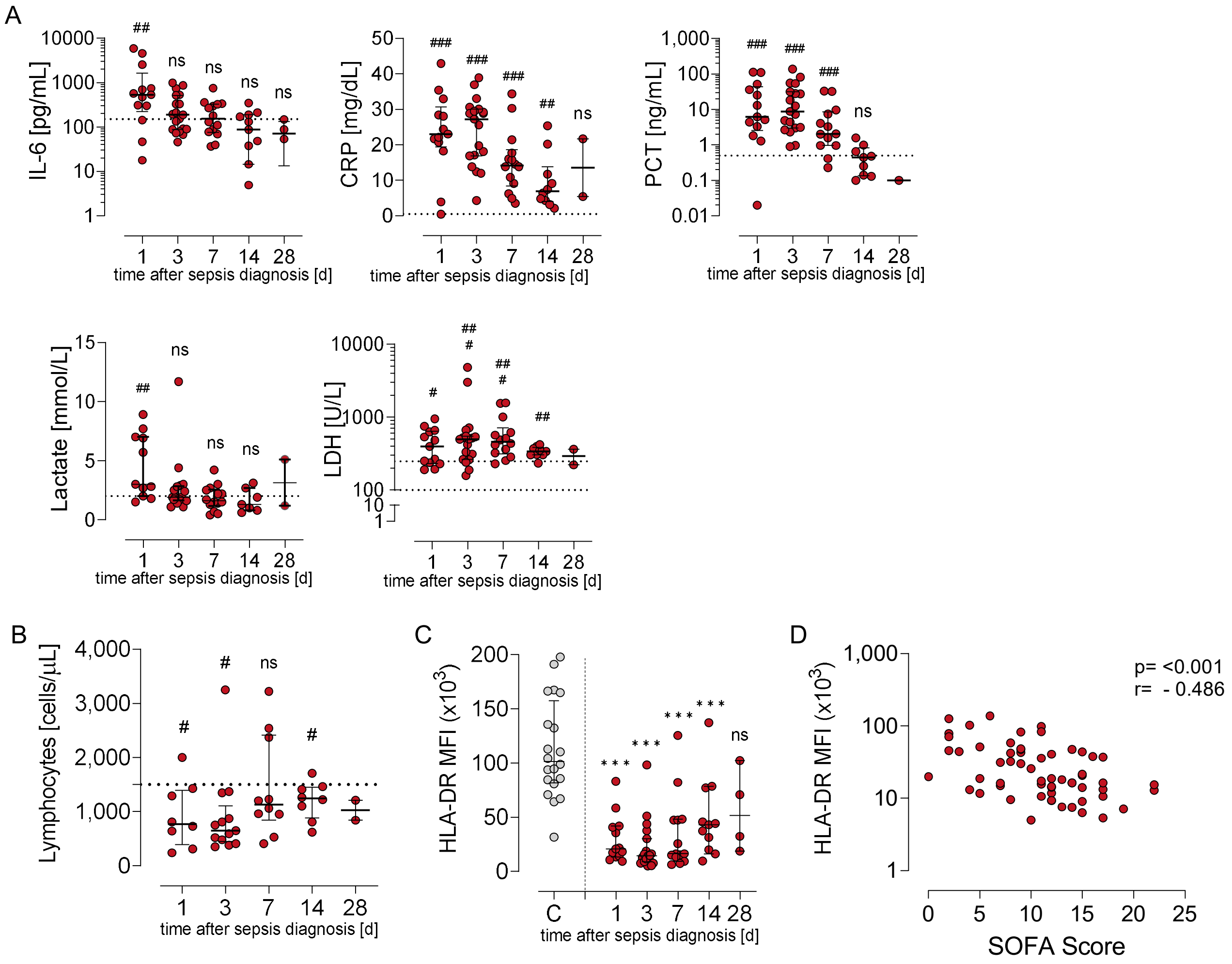

Suppl. Figure 2. Patients display characteristic markers associated with sepsis. (A) Concentration of IL-6, CRP, PCT, Lactate, and LDH in the sera from patients. Dotted lines indicate the reference values. **(B)** Lymphocyte count in the blood of septic patients on day 1, 3, 7, 14, and 28 after sepsis diagnosis. Data in (A; except for IL-6) and (B) were determined during routine diagnostics (n=2-19) where available. **(C)** HLA-DR Expression on gated CD14^{+} monocytes of healthy controls (C) and sepsis patients. **(D)** Spearman correlation between HLA-DR expression and SOFA-Score (n=4-19). Horizontal lines indicate the median/interquartile range of individual values. Statistically significant differences were tested using the Mann-Whitney test (controls vs. patients; ***, p < 0.001), One-sample Wilcoxon test (patients vs. reference value; #, p < 0.05; ##, p < 0.01; ###, p < 0.001) **(A, D)**. CRP. C-reactive protein; PCT, Procalcitonin; LDH, lactate dehydrogenase

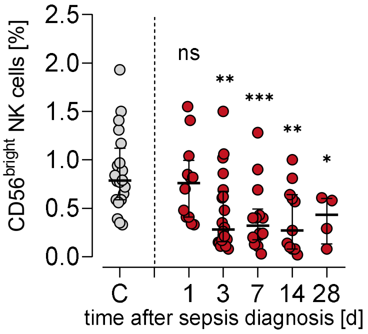

Suppl. Figure 3. Frequency of CD56^{bright} NK cells. PBMCs were isolated from control subjects (C; n=20) and from septic patients on days 1, 3, 7, 14, and 28 after the onset of sepsis (n=4–19) and the percentage of CD56^{bright} NK cells within the lymphocyte gate (see Suppl. Fig. 1) was determined. Statistical differences between controls and septic patients were assessed using Mann-Whitney test (*, $p < 0.05$; **, $p < 0.01$; ***, $p < 0.001$).

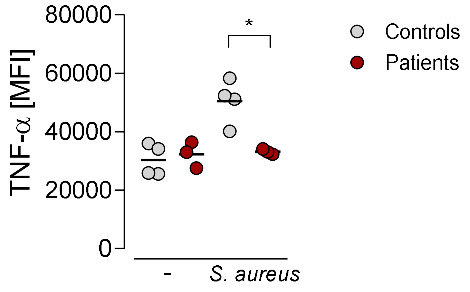

Suppl. Figure 4. **Expression of TNF-α by CD56^{bright} NK cells in response to *S. aureus*.** Frozen PBMCs from control subjects (n=4) and from patients on day 3 (n=3) after sepsis diagnosis were thawed and stimulated with heat-killed *S. aureus* in the presence of autologous serum. The expression of TNF-α by CD56^{bright} NK cells was determined by flow cytometry. Horizontal lines indicate the mean of individual values. Statistical differences between controls (C) and patients were tested using Student's t-test. (*, $p < 0.05$). MFI, median fluorescence intensity

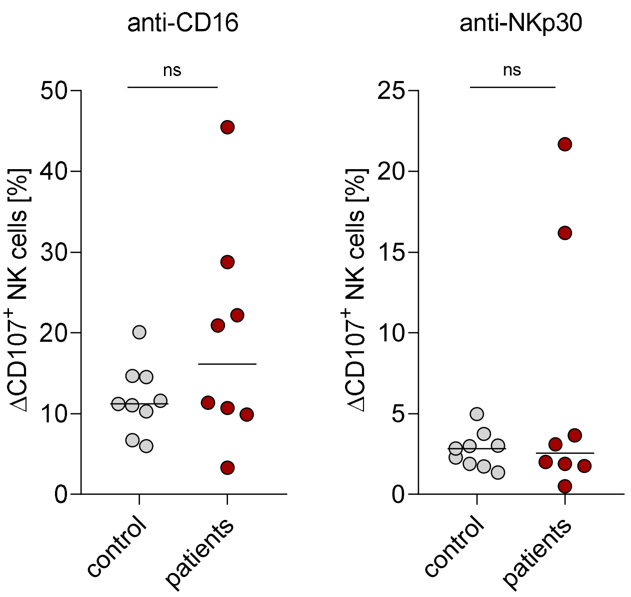

Suppl. Figure 5. **CD16- and NKp30-mediated degranulation of NK cells does not change during sepsis.** Frozen PBMC from control subjects (n=9) and from patients on day 3 after diagnosis of sepsis (n=8) were thawed and stimulated for 3 h with plate-bound antibodies against CD16 (clone 3G8) or NKp30 (clone p30.15). Wells coated with non-specific MOPC21 antibodies served as negative controls. Degranulated NK cells were identified by CD107a externalization and analyzed by flow cytometry. Data are shown as Δ (Stimulation – negative control). Statistical differences were tested using Mann-Whitney test. ns, not significant

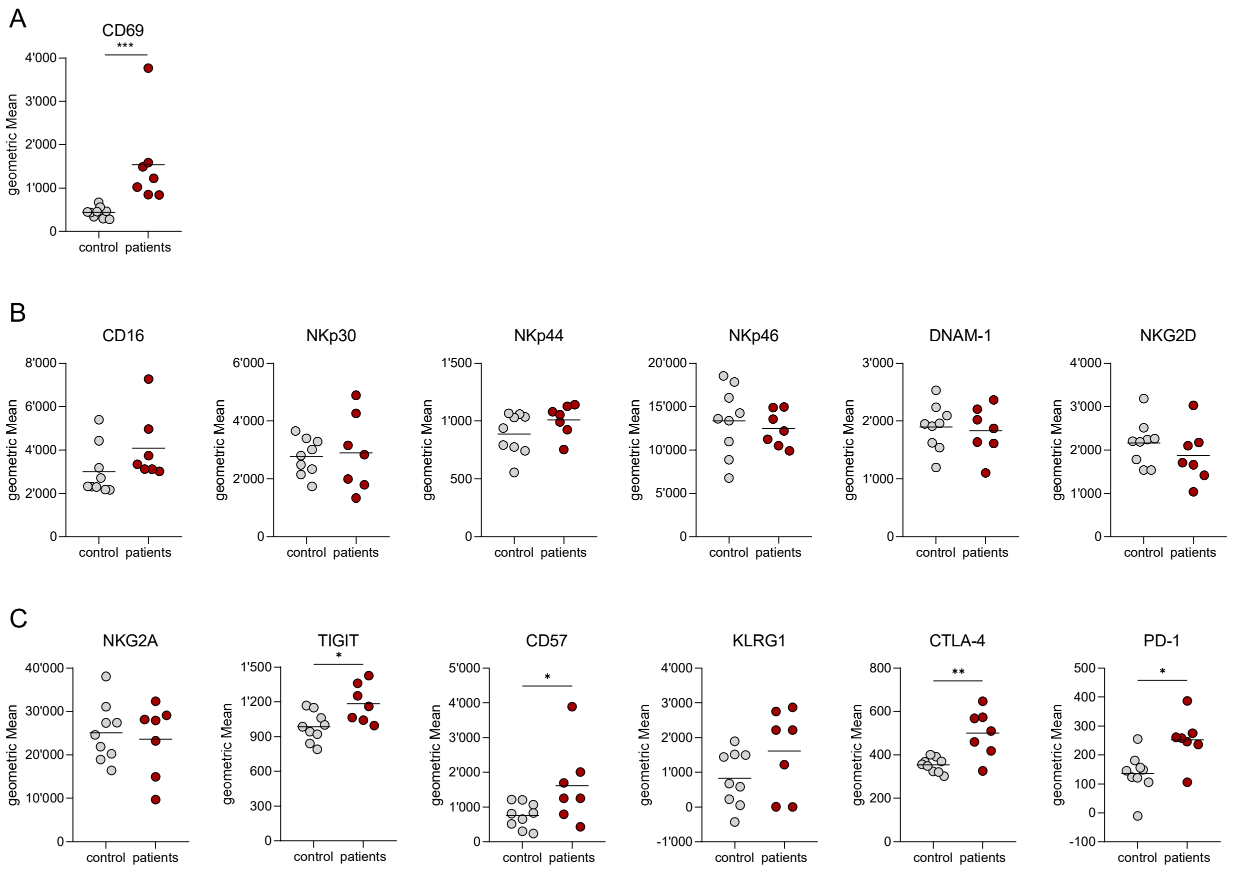

Suppl. Figure 6. **Expression of activation and exhaustion markers, activating and inhibitory receptors on CD56^{bright} NK cells.** Frozen PBMC from control subjects (n=9) and from patients on day 3 after diagnosis of sepsis (n=7) were thawed and stained for **(A)** the activation marker CD69, **(B)** activating receptors and **(C)** inhibitory receptors and exhaustion markers. Cells were analyzed by spectral flow cytometry. Data are shown as mean (horizontal lines) of individual values of the geometric mean of the fluorescence intensity of the indicated markers on CD56^{bright} NK cells. Statistical differences were tested using the Mann-Whitney test (controls vs. patients; *, $p < 0.05$; **, $p < 0.01$; ***, $p < 0.001$).

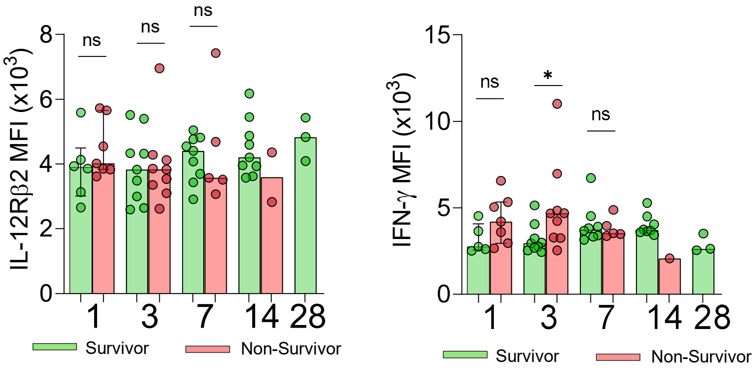

Suppl. Figure 7. **Expression of IL-12Rβ2 and IFN-γ in response to *S. aureus* in survivors and non-survivors.** PBMCs from patients on days 1, 3, 7, 14 and 28 (n=4-19) after sepsis diagnosis were stimulated with heat-killed *S. aureus* in the presence of autologous serum. The expression of IL-12Rβ2 and IFN-γ was determined (overall data shown in Fig. 1B, C) and displayed according to survival and non-survival. Horizontal lines indicate the median/interquartile range of individual values. Statistical differences between survivors and non-survivors were tested using the Mann-Whitney test. (*, $p<0.05$; ns, not significant)

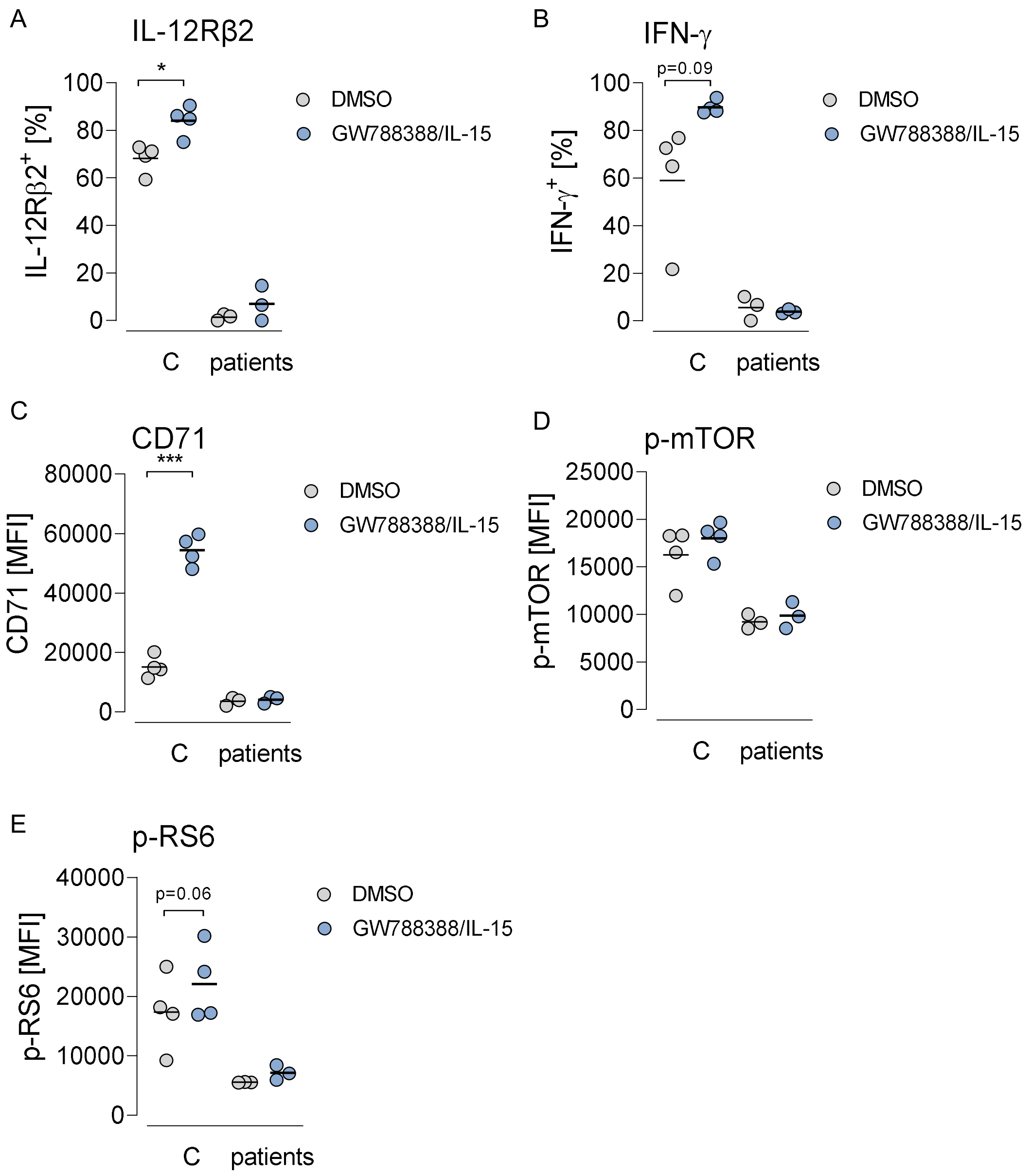

Suppl. Figure 8. Responsiveness of CD56^{bright} NK cells to the ALK5 inhibitor GW788388 and IL-15 during sepsis. Frozen PBMC from control subjects (n=4) and from patients on day 3 after sepsis diagnosis (n=3) were thawed and stimulated with inactivated *S. aureus* each in the absence (DMSO as solvent control) or presence of GW788388 and recombinant IL-15. The expression of IL-12Rβ2, IFN-γ, CD71, p-mTOR, and p-RS6 of CD56^{bright} NK cells was determined by flow cytometry. Horizontal lines indicate the mean of individual values. Statistically significant differences between DMSO and GW788388/IL-15 were tested using paired Student's t-test (*, $p < 0.05$; ***, $p < 0.001$). MFI, median fluorescence intensity

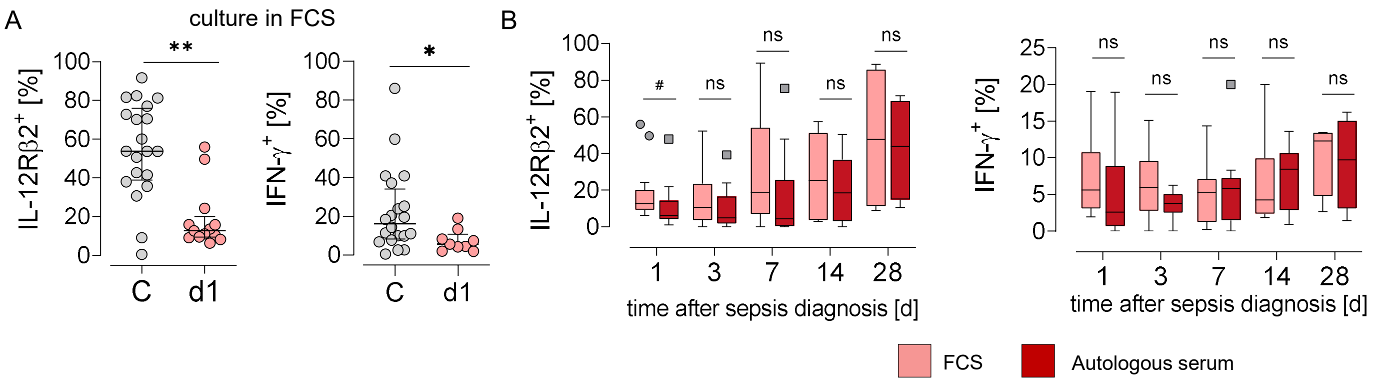

Suppl. Figure 9. The disturbed IL-12R/IFN-γ axis of CD56^{bright} NK cell during sepsis is maintained in the absence of autologous serum. PBMCs from control subjects (C; n=20) and from patients on day 1, 3, 7, 14, and 28 (n=4-19) after diagnosis of sepsis were stimulated with heat-inactivated *S. aureus* in the presence of fetal calf serum (FCS) or autologous serum. The expression of IL-12Rβ2 and IFN-γ on gated CD56^{bright} NK cells was determined by flow cytometry. **(A)** Expression of the IL-12Rβ2 and IFN-γ of CD56^{bright} NK cells from controls and patients on day 1 after sepsis diagnosis. **(B)** Cumulative data on the frequency of IL-12Rβ2^{+} and IFN-γ^{+} NK cells stimulated in the presence of FCS or autologous serum throughout the observation period (data for autologous serum are the same as in Fig. 1B, C). Horizontal lines indicate the median/interquartile range. Statistically significant differences between controls and patients were tested using the Mann-Whitney test (A). Statistically significant differences between culture conditions were tested using the Wilcoxon signed rank test. (*, $p < 0.05$; **, $p < 0.01$; $^{\#}$, $p < 0.05$). ns, not significant

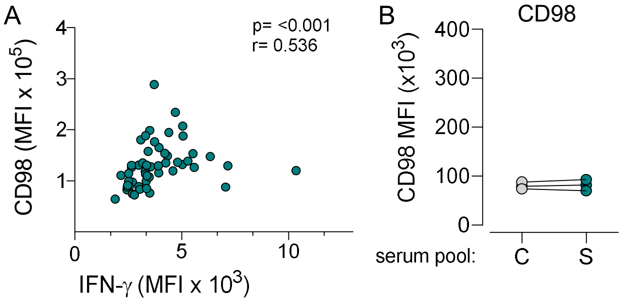

Suppl. Figure 10. Baseline expression of IFN-γ correlates with the expression of the amino acid transporter CD98 on CD56^{bright} NK cells. (A) Spearman correlation between the baseline expression of IFN-γ and CD98 on CD56^{bright} NK cells from septic patients. **(B)** PBMC from healthy donors (n=3) were cultured in medium supplemented with pooled sera from healthy donors (C) or from septic patients (S) obtained 1 d after sepsis diagnosis. The expression of CD98 on CD56^{bright} NK cells was determined. MFI, median fluorescence intensity

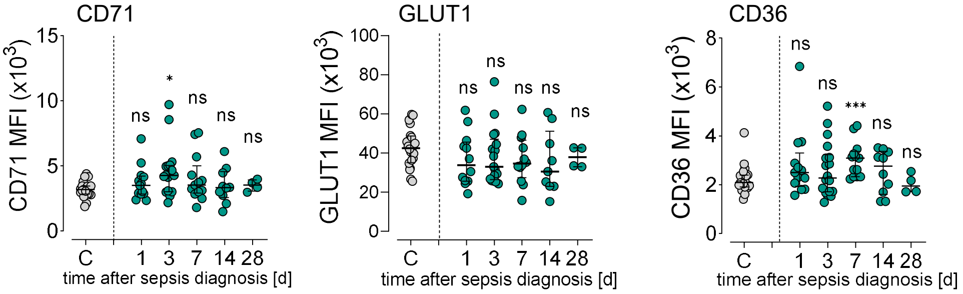

Suppl. Figure 11. Nutrient transporter expression on CD56bright NK cells at baseline during sepsis. PBMC from control subjects (C; n=20) and from patients on day 1, 3, 7, 14, and 28 after sepsis diagnosis (n=4-18) were cultured in medium supplemented with autologous serum. The expression of CD71, Glut1, and CD36 on CD56bright NK cells was determined. Horizontal lines indicate the median/interquartile range of individual values. Statistically significant differences between controls and patients were tested using the Mann-Whitney test (*, $p < 0.05$; ***, $p < 0.001$; ns, not significant). MFI, median fluorescence intensity

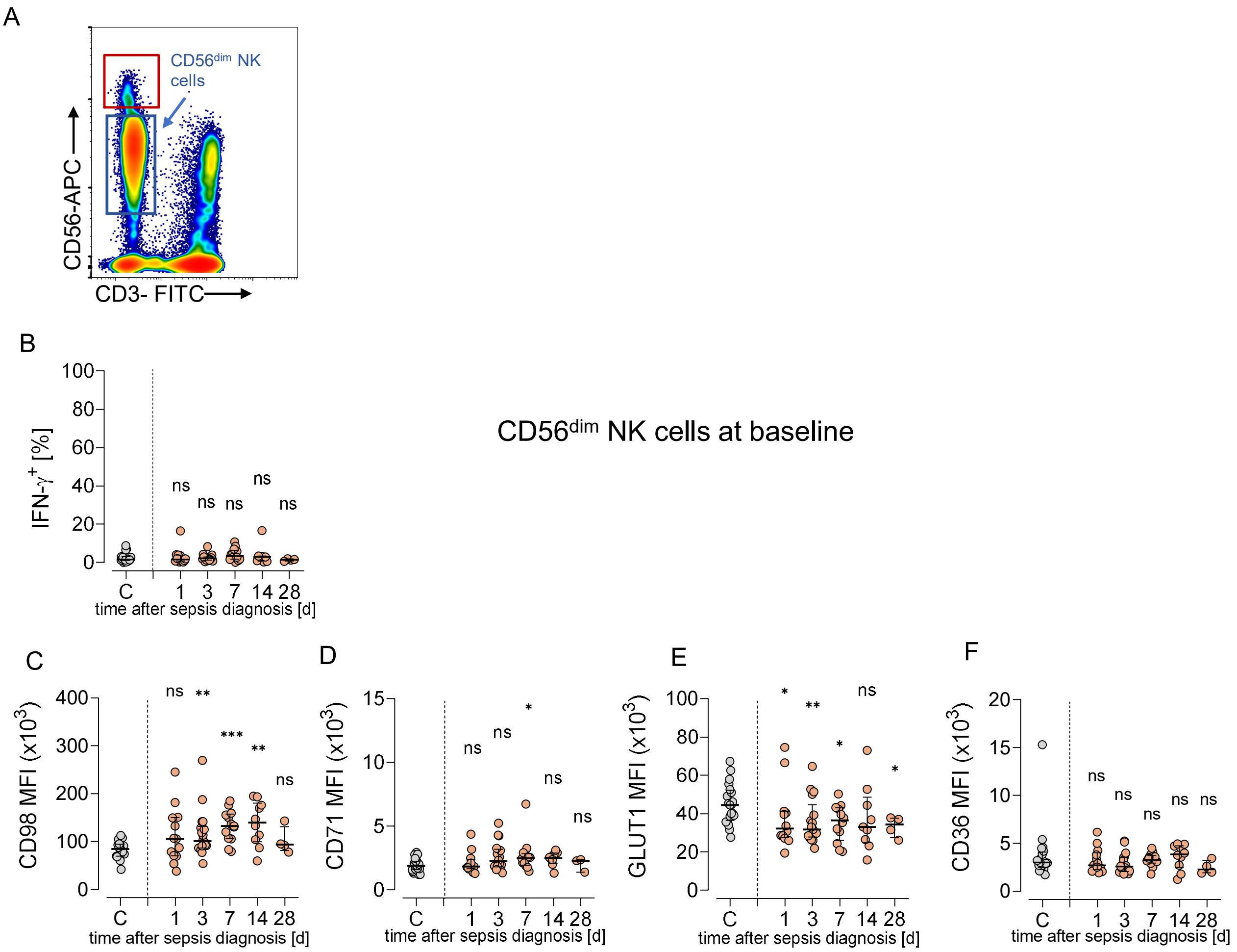

Suppl. Figure 12. **Expression of IFN-γ and nutrient transporters by CD56^{dim} NK cells at baseline.** PBMCs from control subjects and from patients on days 1, 3, 7, 14 and 28 (n=4-19) after sepsis diagnosis were cultured in medium supplemented with autologous serum. **(A)** Gating of CD56^{dim} NK cells. Expression of **(B)** IFN-γ, **(C)** CD98, **(D)** CD71, **(E)** GLUT1, **(F)** CD36 on CD56^{dim} NK cells was determined. Horizontal lines indicate the median/interquartile range of individual values. Statistical differences between controls (C) and patients were tested using the Mann-Whitney test. (*p < 0.05, **p < 0.01, ***p < 0.001, ns, not significant)

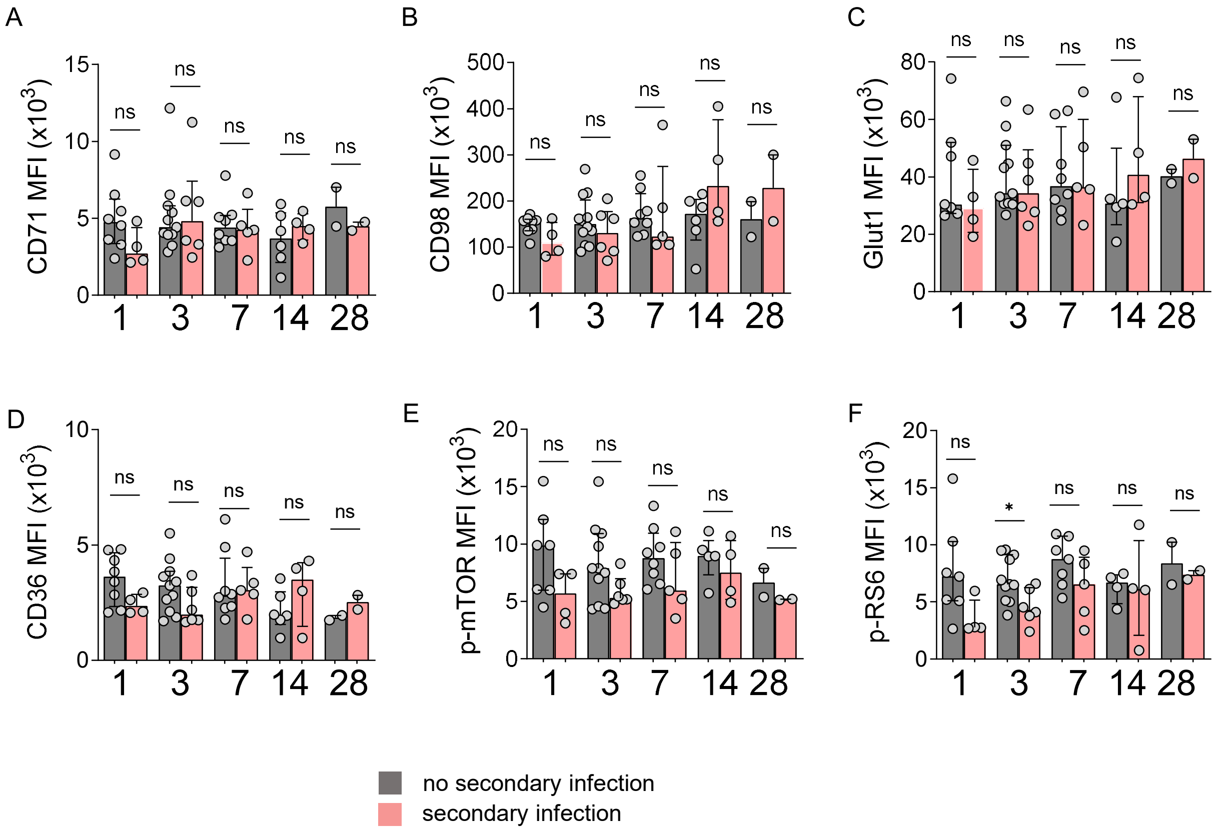

Suppl. Figure 13. **Expression of nutrient transporters and activation of mTORC1 by CD56^{bright} NK cells in response to *S. aureus* in association with the development of secondary infection.** PBMCs from patients on days 1, 3, 7, 14 and 28 (n=4-19) after sepsis diagnosis were stimulated with heat-killed *S. aureus* in the presence of autologous serum. The expression of **(A-D)** CD71, CD98, Glut1, CD36, and **(E, F)** mTORC1 (mTOR and pS6) was determined (overall data shown in Fig. 5D and Fig. 6C) and displayed according to the development of secondary infection. Horizontal lines indicate the median/interquartile range of individual values. Statistical differences between development of secondary infections and free of secondary infection were tested using the Mann-Whitney test. (*, $p < 0.05$; ns, not significant)

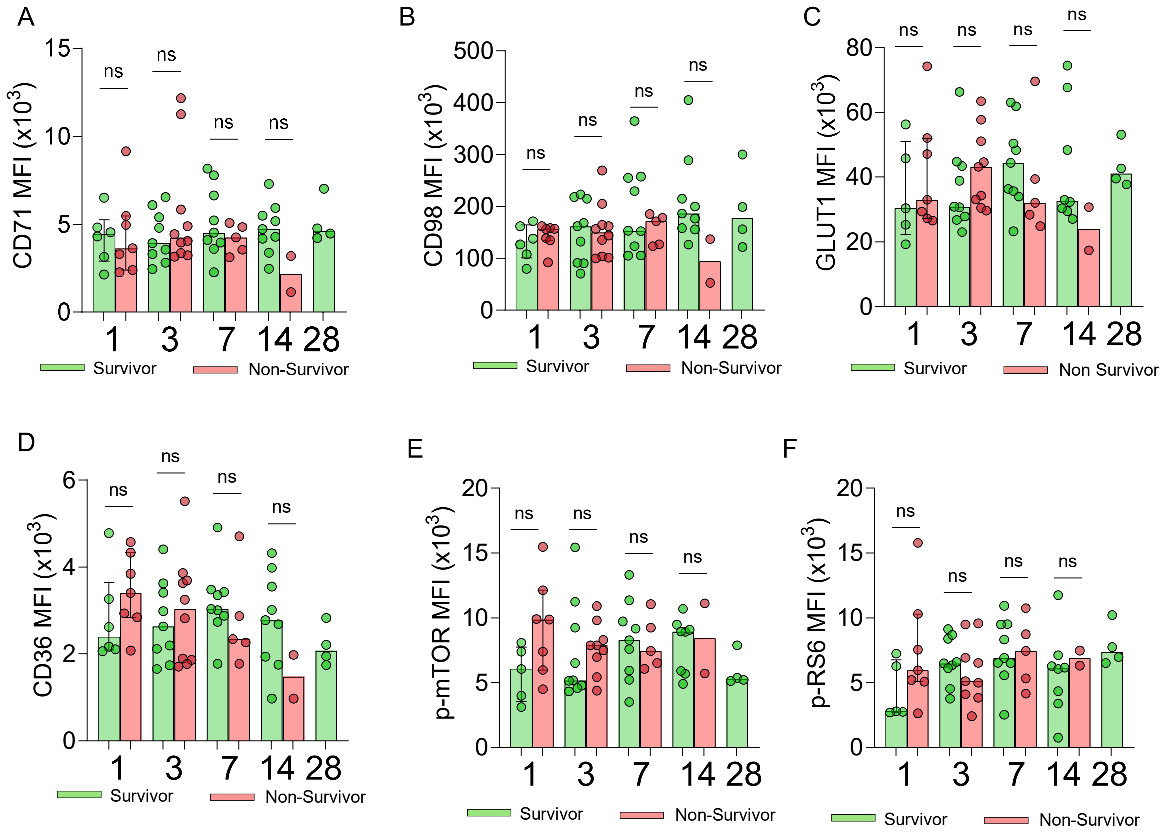

Suppl. Figure 14. **Expression of nutrient transporters and activation of mTORC1 in response to *S. aureus* in survivors and non-survivors.** PBMCs from patients on days 1, 3, 7, 14 and 28 (n=4-19) after sepsis diagnosis were stimulated with heat-killed *S. aureus* in the presence of autologous serum. The expression of **(A-D)** CD71, CD98, Glut1, CD36, and **(E, F)** mTORC1 (mTOR and pS6) was determined (overall data shown in Fig. 5D and Fig. 6C) and displayed according to survival and non-survival. Horizontal lines indicate the median/interquartile range of individual values. Statistical differences between survivors and non-survivors were tested using the Mann-Whitney test. (ns, not significant)

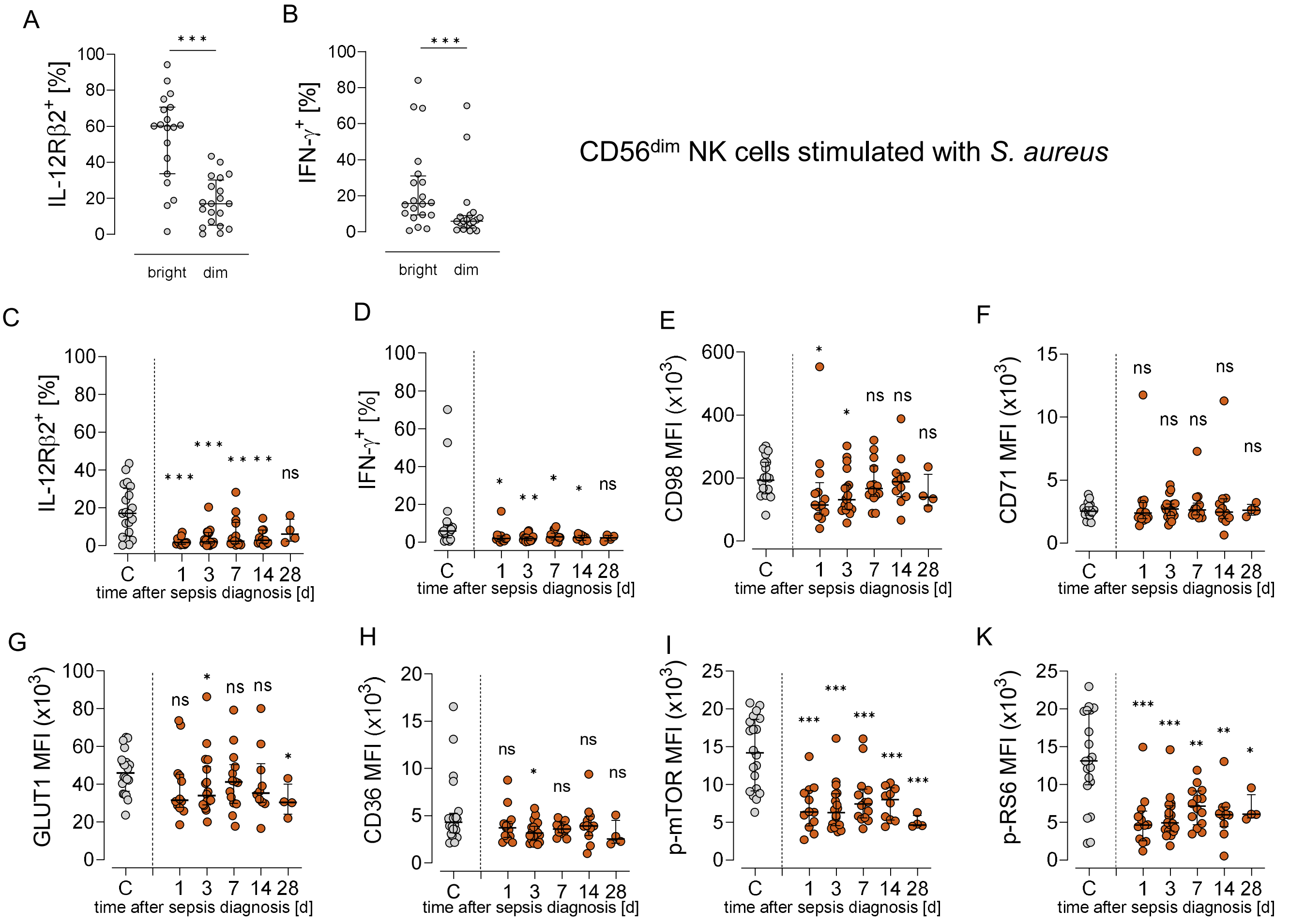

Suppl. Figure 15. **Expression of IL-12Rβ2, IFN-γ, nutrient transporters and activation of mTORC1 by CD56^{dim} NK cells in response to *S. aureus*.** PBMCs from control subjects and from patients on days 1, 3, 7, 14 and 28 (n=4-19) after sepsis diagnosis were stimulated with heat-killed *S. aureus* in the presence of autologous serum. Comparison of the expression of **(A)** IL-12Rβ2 and **(B)** IFN-γ between CD56^{bright} and CD56^{dim} NK cells of control subjects . The expression of **(C)** IL-12Rβ2, **(D)** IFN-γ, **(E)** CD98, **(F)** CD71, **(G)** Glut1, **(H)** CD36, **(I)** p-mTORC, and **(K)** p-RS6 on CD56^{dim} NK cells of control subjects and septic patients was determined. Horizontal lines indicate the median/interquartile range of individual values. Statistical differences between controls (C) and patients were tested using the Mann-Whitney test. (*, $p < 0.05$; **, $p < 0.01$; ***, $p < 0.001$; ns, not significant)

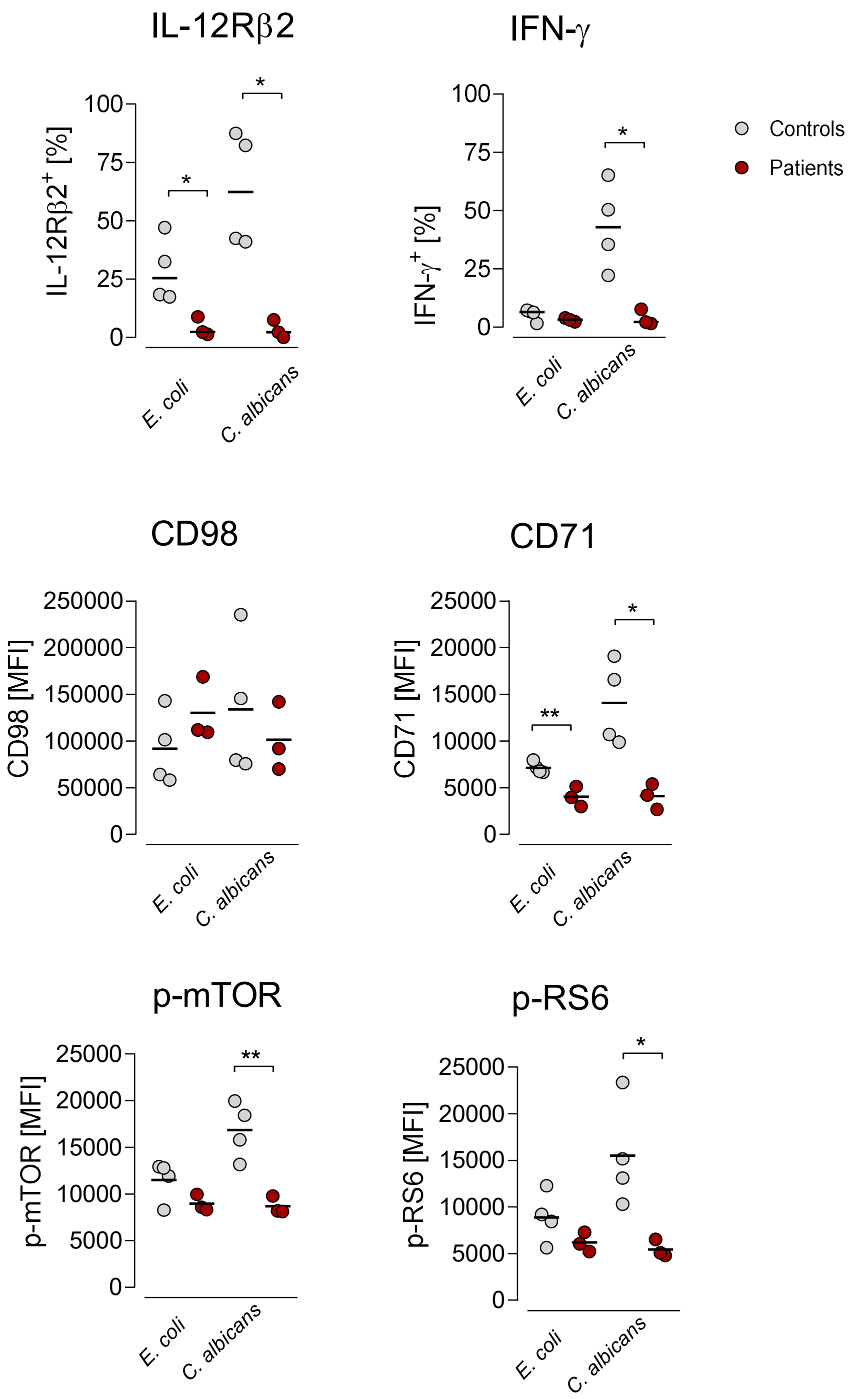

Suppl. Figure 16. Responsiveness of CD56^{bright} NK cells to *E. coli* and *Candida albicans* during sepsis. Frozen PBMC from control subjects (n=4) and from patients on day 3 after sepsis diagnosis (n=3) were thawed and stimulated with inactivated *E. coli* or *C. albicans*. The expression of IL-12Rβ2, IFN-γ, CD98, CD71, p-mTOR, and p-RS6 of CD56^{bright} NK cells was determined by flow cytometry. Horizontal lines indicate the mean of individual values. Statistically significant differences between controls and patients were tested using unpaired Student's t-test (*, $p < 0.05$; **, $p < 0.01$). MFI, median fluorescence intensity
