# Supplementary material for: Disturbed metabolic adaptation drives natural killer cell dysfunction in association with nosocomial infection during human sepsis
Source: eBioMedicine. 2026 Jun 26;129:106345. doi: 10.1016/j.ebiom.2026.106345 (PMC13324300; doi:10.1016/j.ebiom.2026.106345)
Supplement: Supplemental Materials and Methods [file mmc2.pdf]

Supplemental Materials and Methods

Spectral flow cytometry of surface receptors on NK cells

All antibodies were individually titrated to determine the optimal dilution. All reagents, antibodies and dilutions are listed in Suppl. Table 2. PBMC were used immediately after thawing. For each sample, cells were stained with the live/dead stain Zombie NIR in PBS for 20 min in the dark at RT and washed with FACS buffer (PBS / 2% FCS). Afterwards, samples were stained with 50 µl of the antibody cocktail for 20 min at 4 °C in the dark and then washed with FACS buffer. Cells were resuspended in 150 µl FACS buffer and kept at 4 °C until analysis at the same day on a 5 laser Cytek® Aurora spectral flow cytometer (Cytek® Biosciences). Data were analyzed using the FlowJo™software (version 10.10; BD Life Sciences, USA). Statistical analyses were performed with GraphPad Prism 10 (GraphPad software LLC).

Degranulation assay

All antibodies were individually titrated to determine the optimal dilution. All reagents, antibodies and dilutions are listed in Suppl. Table 1. PBMC were thawed and resuspended in RPMI supplemented with 10 % FCS and 1 % penicillin/streptomycin (all from Gibco, Life Technologies). Cells were rested for 3 h in a humidified incubator at 37 °C and 5 % CO_2 and then distributed in a 96 well Maxisorp (Nunc) flat bottom plate coated with antibodies against CD16 (3G8) or NKp30 (p30.15). Wells coated with non-specific MOPC21 served as controls. Cells were incubated for 3 h in a humidified incubator at 37 °C and 5 % CO_2, and then transferred to a 96 well V bottom plate. Cells were stained for CD56, CD3 and CD107a in FACS buffer for 20 min at 4 °C in the dark. Afterwards, cells were washed, resuspended in 150 µl FACS buffer and kept at 4 °C until analysis at the same day on a 5 laser Cytek® Aurora spectral flow cytometer (Cytek® Biosciences). Data were analyzed using the FlowJo™software (version 10.10; BD Life Sciences, USA). Statistical analyses were performed with GraphPad Prism 10 (GraphPad software LLC).

Supplemental Tables

Suppl. Table 1. Antigens and antibody clones used for functional assays

Target	clone	Fluorochrome	Catalog No	distributor	dilution 1/x
CD3	OKT-3	FITC	21850033	Immunotools	100
CD56	CMSSB	APC	17-0567-42	Thermo Fisher Scientific	40
IL12Rβ2	REA333	PE	130-120-068	Miltenyi Biotec	100
CD71	CY1G4	PE/Cy7	334112	Biolegend	80
CD3	UCHT1	APC A700	300424	BioLegend	50
CD98	REA387	PE	130-120-051	Miltenyi Biotec	100
CD36	5-271	FITC	336204	BioLegend	25
CD56	CMSSB	APC	17-0567-42	Thermo Fisher Scientific	20
IFN-ɣ	4S.B3	PE	502509	BioLegend	80
mTOR	MRRBY	PE	12-9718-42	Thermo Fisher Scientific	100
RPS6	A17020B	BV421	608610	BioLegend	22,2
GLUT1	EPR3915	APC A700	ab195359	Abcam	500
live/dead	n/a	Zombie Aqua	423102	Biolegend	133
**Reagents**					
GolgiStop			554724	BD Biosciences	100
Cell Wash			349524	BD Bioscience	
Perm/Wash Buffer			51-2091KZ	BD Bioscience	
CytoPerm/Fix			51-2090KZ	BD Bioscience	
FoxP3/Transcription Factor Staining Buffer Set			00-5523-00	Thermo Fisher Scientific	

Suppl. Table 2. Antigens and antibody clones used for phenotyping and degranulation assays

Target	clone	Fluorochrome	Catalog No	distributor	dilution 1/x
**Phenotyping**					
CD3	UCHT1	BUV563	748569	BD Biosciences	200
CD16	3G8	BUV615	751572	BD Biosciences	500
CD56	B159	BUV805	742022	BD Biosciences	200
CD335 (NKp46)	9E2	BV421	331914	Biolegend	50
CD57	TB01	eFluor450	48-0577-42	Thermo Fisher Scientific	200
KLRG1	2F1	BV510	138421	Biolegend	50
CD45	HI30	BV570	304034	Biolegend	400
CD336 (NKp44)	p44-8	BV711	744303	BD Biosciences	200
CD69	FN50	BV750	310954	Biolegend	400
TIGIT	741182	BV786	747838	BD Biosciences	100
CD226 (DNAM-1)	DX11	FITC	559788	BD Biosciences	100
CD159a (NKG2A)	Z199	PE	IM3291U	Beckman Coulter	100
CD152 (CTLA-4)	BNI3	PE-Dazzle	369616	Biolegend	100
CD279 (PD-1)	EH12.1	AF647	560838	BD Biosciences	100
CD314 (NKG2D)	FAB139N	AF700	FAB139N	R&D Systems	100
live / dead	n/a	Zombie NIR	423106	Biolegend	700
CD337 (NKp30)	p30.15	APC-Fire 750	325226	Biolegend	100
**Degranulation**					
CD3	UCHT1	BUV563	748569	BD Biosciences	200
CD56	B159	BUV805	742022	BD Biosciences	200
CD107a (LAMP-1)	H4A3	PE-Cy5	560947	BD Biosciences	50
**Reagents**					
True-Stain Monocyte Blocker™			426103	Biolegend	
Brilliant Stain Buffer			563794	BD Biosciences	
Bovine serum			10270-106	Thermo Fisher Scientific	

Suppl. Table 3. Causative pathogens of primary (sepsis-inducing) and secondary infection

Parameter	Patients (n=20)
Microbiologicially documented sepsis-inducing pathogens, n (%)	
Staphylococcus aureus	4 (20%)
Coagulase-negative *Staphylococcus spp.*	5 (25%)
Enterococcus faecium	1 (5%)
Escherichia coli	3 (15%)
Klebsiella pneumoniae	1 (5%)
Pseudomonas aeruginosa	1 (10%)
Bacteroides fragilis	1 (5%)
Candida spp.	3 (15%)
Microbiologicially documented pathogens inducing secondary infection after sepsis diagnosis, n (%)	
Pseudomonas aeruginosa	1 (17%)
Klebsiella pneumoniae	1 (17%)
Candida spp.	2 (32%)
Aspergillus spp.	1 (17%)
Herpes simplex virus	1 (17%)
