# Supplementary material for: Disturbed metabolic adaptation drives natural killer cell dysfunction in association with nosocomial infection during human sepsis
Source: eBioMedicine. 2026 Jun 26;129:106345. doi: 10.1016/j.ebiom.2026.106345 (PMC13324300; doi:10.1016/j.ebiom.2026.106345)
Supplement: Reagents antibodies [file mmc3.pdf]

Suppl. Table 1. Reagents, kits, and antibodies

**Reagent/substance**	**Company**	**Catalogue no.**
Lithium-Heparin Monovetten	Sarstedt	01.1604
Serum S-Monovette Z;clot activator	Sarstedt	02.1063
Ficoll Paque Plus	Cytiva/GE Healthcare	17144003
Red Blood Cell Lysis Solution	Miltenyi Biotec	130-094-183
MACSxpress Whole blood NK cell isolation kit	Miltenyi Biotec	130-127-695
VLE RPMI 1640 medium	PAN-Biotech	P04-18525
Penicillin/ Streptomycin	Gibco Life Technologies	15140-122
FCS	Biochrom	S0115
SB431542	Tocris	1614
recombinant IL-15	PeproTech	AF-200-15
recombinant IL-12	Biolegend	573002
recombinant IL-18	MBL	B001-5
Compound C	BioGems	8666430
DMF	Roth	6251
heat-inactivated *S. aureus*	Invivogen	tlrl-hksa
heat-inactivated *E. coli*	Invivogen	tlrl-hkeb2
Candida albicans	Invivogen	tlrl-hkca
IL-6 DuoSet Elisa	R&D Systems, Biotechne	DY206
GDF-15 DuoSet Elisa	R&D Systems, Biotechne	DY957
GolgiStop	554724	BD Biosciences
Cell Wash	349524	BD Bioscience
Perm/Wash Buffer	51-2091KZ	BD Bioscience
CytoPerm/Fix	51-2090KZ	BD Bioscience
FoxP3/Transcription Factor Staining Buffer Set	00-5523-00	eBioscience

Target	clone	Fluorochrome	Catalogue no.	Company	dilution 1/x
CD3	MEM-57	FITC	21270033	Immunotools	100
CD3	OKT-3	FITC	21850033	Immunotools	100
CD56	CMSSB	APC	AB_10597454	eBioscience	40
IL12Rβ2	REA333	PE	AB_2751988	Miltenyi Biotec	100
CD71	CY1G4	PE/Cy7	AB_2563119	Biolegend	80
CD3	UCHT1	APC A700	AB_493741	BioLegend	50
CD98	REA387	PE	AB_2751975	Miltenyi Biotec	100
CD36	5-271	FITC	AB_1575025	BioLegend	25
CD56	CMSSB	APC	AB_10597454	eBioscience	20
IFN-g	4S.B3	PE	AB_315234	BioLegend	80
mTOR	MRRBY	PE	AB_2572724	eBioscience	100
RPS6	A17020B	BV421	AB_2814451	BioLegend	22,2
GLUT1	EPR3915	APC A700	AB_2714026	Abcam	500
live/dead	n/a	Zombie Aqua	423102	Biolegend	133
